# Immune responses, therapeutic anti-tumor effects, and tolerability upon therapeutic HPV16/18 E6/E7 DNA vaccination via needle-free biojector

**DOI:** 10.1128/mbio.02121-23

**Published:** 2023-10-04

**Authors:** Shiwen Peng, Hsin-Fang Tu, Michelle Cheng, Ming-Hung Hu, Hua-Ling Tsai, Ya-Chea Tsai, Chelsea Koenig, Cory Brayton, Hao Wang, Yung-Nien Chang, Rebecca C. Arend, Kimberly Levinson, Richard B. S. Roden, T. C. Wu, Chien-Fu Hung

**Affiliations:** 1 Department of Pathology, Johns Hopkins University, Baltimore, Maryland, USA; 2 Department of Oncology Biostatistics, Johns Hopkins University, Baltimore, Maryland, USA; 3 Department of Molecular and Comparative Pathobiology, Johns Hopkins University, Baltimore, Maryland, USA; 4 Papivax Biotech Inc,, Taipei, Taiwan ROC; 5 Department of Obstetrics and Gynecology, O’Neal Comprehensive Cancer Center, University of Alabama at Birmingham, Birmingham, Alabama, USA; 6 Department of Oncology, Johns Hopkins University, Baltimore, Maryland, USA; 7 Department of Obstetrics and Gynecology, Johns Hopkins University, Baltimore, Maryland, USA; Harvard Medical School, Boston, Massachusetts, USA

**Keywords:** human papillomavirus, HPV16, HPV18, E6, E7, DNA vaccine, HPV-associated cancers, needle-free biojector, customized needle-free tropis system

## Abstract

Intramuscular vaccination of mice with the naked pBI-11 DNA plasmid targeting E6 and E7 of HPV16 and HPV18 via a conventional syringe and needle generates human papillomavirus (HPV) antigen-specific CD8+ T cell-mediated immune responses and therapeutic effects against the TC-1 tumor model. However, delivery of DNA vaccines by this method is less effective in patients, likely due to poor transduction of host tissues. Needle-free biojectors show great promise for DNA vaccination because of their simplicity of administration and high patient acceptability and also, we hypothesize, because of greater efficiency of cell transduction in host tissues. Here, we compared the kinetics of transgene expression from a plasmid DNA using intramuscular injection with a conventional needle administration to intradermal or intramuscular delivery with a customized Tropis biojector. Delivery using the customized Tropis biojector leads to enhanced transgene expression compared to intramuscular needle injection. In addition, we characterized the HPV antigen-specific CD8+ T cell-mediated immune responses and anti-tumor effects generated by pBI-11 DNA vaccination by each route of administration, as well as by split-dose multi-site injection. Intradermal, but not intramuscular, vaccination with pBI-11 DNA vaccine via customized Tropis biojector enhanced HPV antigen-specific CD8+ T cell-mediated immune responses over needle injection. Intradermal, but not intramuscular, vaccination via customized needle-free Tropis biojector elicited greater HPV antigen-specific CD8+ T cell-mediated immune responses as well as anti-tumor effects compared to intramuscular injection of pBI-11 with a needle. Good manufacturing practices (GMP) grade pBI-11 DNA vaccine delivered intradermally or intramuscularly via customized Tropis biojector was well tolerated by mice.

**IMPORTANCE:**

Respectively, HPV16 and HPV18 cause 50% and 20% of cervical cancer cases globally. Viral proteins E6 and E7 are obligate drivers of oncogenic transformation. We recently developed a candidate therapeutic DNA vaccine, pBI-11, that targets HPV16 and HPV18 E6 and E7. Single-site intramuscular delivery of pBI-11 via a needle elicited therapeutic anti-tumor effects in mice and is now being tested in high-risk human papillomavirus+ head and neck cancer patients (NCT05799144). Needle-free biojectors such as the Tropis device show promise due to ease of administration, high patient acceptability, and the possibility of improved delivery. For example, vaccination of patients with the ZyCoV-D DNA vaccine using the Tropis device is effective against COVID19, well tolerated, and licensed. Here we show that split-dose, multi-site administration and intradermal delivery via the Tropis biojector increase the delivery of pBI-11 DNA vaccine, enhance HPV antigen-specific CD8+ T-cell responses, and improve anti-tumor therapeutic effects, suggesting its translational potential to treat HPV16/18 infection and disease.

## INTRODUCTION

Human papillomavirus (HPV) is a known cause of 5% of cancer worldwide, including cervical, vulvar, vaginal, anal, and head and neck cancers (1). Of over 200 identified genotypes, only a dozen types are considered high-risk human papillomavirus (hrHPV) for carcinogenic capacity (1). Over 95% of cervical cancer is attributed to hrHPV infection, with HPV16 and HPV18 being responsible for 50% and 20% of cases, respectively (1, 2). Therefore, vaccines targeting hrHPV have been developed to prevent cancer and its precursors driven by these genotypes. Gardasil and Cervarix are two widely used preventative HPV vaccines that contain empty virus-like particles (including those derived from HPV16 and HPV18), but unfortunately, they are not effective for treating established infection and its associated diseases. Furthermore, significant portions of the population remain unvaccinated against HPV, and around 85% of those who are unvaccinated will get an HPV infection (3). Fortunately, population-based Pap and/or HPV screening is used in many countries, and this creates an opportunity for virus-specific treatment interventions.

The control of existing HPV infections and/or HPV-associated disease requires a different approach than preventative HPV vaccines. While preventative vaccines target the viral capsid with neutralizing antibodies, therapeutic vaccines will likely require targeting HPV early proteins with CD8 T cell-based cellular immunity because these early proteins are only present inside infected cells (4).

For three main reasons, therapeutic vaccines (immunotherapies) being developed for the treatment of HPV-associated cancers commonly target E6 and E7 (4). First, the E6/E7 oncogenic proteins are expressed in all HPV-infected cells and malignancies and are absent in healthy cells, providing a lesion-specific therapeutic target (5, 6). Additionally, E6/E7 play an essential role in initiating and sustaining HPV-related transformation, preventing their elimination for immune escape (7). Finally, E6/E7 are foreign viral antigens and thus are not subject to central tolerance (8).

Among many possible immunization strategies, DNA vaccines a promising option due to their stability, safety, relative simplicity of manufacturing, as well as their ability to be administered repeatedly. However, the immune response generated by intramuscular (I.M.) injection of naked DNA vaccines is limited due to the low transfection efficiency with needle-based injection and because DNA constructs cannot amplify in cells and spread like a viral vector. Therefore, a better approach is needed to enhance DNA vaccine potency. We have previously created a DNA construct encoding *Mycobacterium tuberculosis* heat shock protein 70 (HSP70) linked to HPV16 E7 antigen and demonstrated that this fusion profoundly enhances the HPV-antigen-specific CD8+ T cell mediated immune response to I.M. DNA vaccination (9). More recently, we have developed the pBI-11 DNA vaccine, in which HPV16/18 E6/E7 antigens are linked to a secretion signal and HSP70 in a single transgene (10). In addition, pBI-11 employs codon optimization of the HPV16/18 E6/E7 genes as a strategy to increase the expression of this fusion protein and enhance the immunogenicity of this DNA vaccine (10). Importantly, pBI-11 DNA vaccine induces HPV16/18 E6/E7-specific CD8+ T-cell immune responses and generates stronger therapeutic responses for mice bearing TC-1 tumor (11). In addition, GMP grade pBI-11 DNA vaccination was well-tolerated by mice (11), and is being tested in patients with advanced hrHPV+ head and neck cancer (NCT05799144).

To maximize the impact of pBI-11 DNA vaccination, it is crucial to have a low cost, simple and highly effective delivery in a manner acceptable to patients globally. One simple approach is to split the dose and inject it into multiple different limbs, which results in a small dose-sparing effect in mice (12) and patients (13). There has been growing interest in needle-free biojectors because of their ease of use, high patient compliance, and feasible application for mass vaccination even in low-resource settings (14, 15). PharmaJet’s Tropis and Stratis injectors are spring-propelled, needle-free systems that deliver vaccines with a narrow, high-velocity fluid jet (16). Tropis has been used for clinical studies and has been shown to induce equal immunogenicity and injection quality when compared to other intradermal (I.D.) methods, with no identified safety concerns (17). Currently, studies investigating the effectiveness and safety of needle-free systems have yielded compelling results with DNA vaccines. DNA vaccines targeting Zika virus as well as SARS-CoV-2 have been safely delivered by the Tropis system and induced neutralizing antibodies in patients (13, 18). Furthermore, SARS-CoV-2 DNA vaccine (ZyCov-D) delivered I.D. by the Tropis system has been approved by Emergency Use Authorization (EUA) in India (19). The Stratis system delivers 0.5 mL of medication I.M., whereas 0.1 mL is delivered I.D. by Tropis (13). The data suggest that DNA delivery through the Tropis/Stratis system is a potentially promising approach for enhancing DNA-based HPV vaccine potency.

In the current study, we compared the immunogenicity and therapeutic anti-tumor effects generated by pBI-11 DNA delivered through I.M. injection with conventional needle and syringe to pBI-11 DNA delivered through I.M. or I.D. injection with a needle-free Tropis system customized with a lower pressure and volume of delivery appropriate for use in mice. In addition, we confirmed the enhancement of the immunogenicity of pBI-11 vaccination by split-dose, multi-site delivery, an approach which was previously found to improve vaccination with other DNA vaccines (12, 13). We found that pBI-11 DNA delivered by the customized needle-free biojector is well tolerated and generates robust HPV antigen-specific CD8+ T-cell immune responses. Intradermal delivery via customized needle-free biojector could elicit a better therapeutic anti-tumor effect as compared to pBI-11 delivered intramuscularly by needle and syringe. In contrast, intramuscular delivery via customized needle-free biojector elicited a similar therapeutic anti-tumor effect as compared to pBI-11 delivered by needle and syringe. Our study has relevance for future clinical translation.

## MATERIALS AND METHODS

### Mice

Female C57BL/6 mice (C57BL/6NTac) were purchased from Taconic Biosciences (Germantown, NY), and housed at the Johns Hopkins University Sidney Kimmel Comprehensive Cancer Center of Johns Hopkins University School of Medicine (Baltimore, MD) animal facility under specific-pathogen free conditions.

### Peptides, antibodies, and other reagents

Peptides of HPV16, E6 aa50-57, YDFAFRDL, HPV16 E7aa49-57, and RAHYNIVTF, and HPV18, E6aa67-75 and KCIDFYSRI, were synthesized by GenScript (Piscataway, NJ) at a purity of ≥80%. BV-780 conjugated anti-mouse CD3 (clone 17A2), fluorescein isothiocyanate (FITC)-conjugated anti-mouse CD4 (clone RM4-5), FITC, or PE, or BV-421-conjugated anti-mouse CD8a (clone 53.6.7), FITC-conjugated anti-mouse interferon gamma (IFN-γ) (clone XMG1.2), APC-A700 conjugated anti-mouse CD44 (clone IM7), PE-conjugated anti-mouse CD62L (clone W18021D) antibodies, and 7-aminoactinomycin D (7-AAD) were purchased from BioLegend (San Diego, CA). PE-conjugated anti-mouse Foxp3 (clone FJK-16s), FITC-conjugated anti-mouse Gr-1 (Ly-6G) (cloneRB6.8C5), and PE-conjugated anti-mouse CD11b (clone M1/70) antibodies were purchased from eBioscience (San Diego, CA). Purified rat anti-mouse CD16/32 (clone 2.4G2) were purchased from Bio X Cell (West Lebanon, NH). PE-conjugated, HPV16 E7aa49-57 peptide-loaded H-2D^b^ tetramers were purchased from MBL International (Japan). Bovine serum albumin (BSA) was purchased from Sigma (St. Louis, MO). Luciferin substrate was obtained from Xenogen Corp. (Alameda, CA). GolgiPlug protein transport inhibitor (containing brefeldin A) was purchased from BD Pharmingen (San Diego, CA). Intracellular fixation and permeabilization buffer set was purchased from eBioscience. Insulin syringes with an ultra-fine needle were purchased from BD Bioscience (NDC/HRI #08290–3284-38; Franklin Lakes, NJ)

### Cell lines

An HPV16 E6- and E7-expressing tumor cell line derived from C57BL/6 mice, TC-1, was established as previously described (20). The TC-1 tumor cell is derived from the C57BL/6 mice and has been used as the preclinical tumor model for the testing of therapeutic HPV vaccine targeting HPV16 E6/E7 antigens. The cells were maintained in RPMI medium supplemented with 2-mM glutamine, 1-mM sodium pyruvate, 100-IU/mL penicillin, 100-µg/mL streptomycin, and 10% fetal bovine serum.

### Vaccine and DNA plasmid

Generation of the DNA vaccine, pBI-11, was described previously (11). The vaccine was prepared and vialed under current good manufacturing practice (cGMP) by Waisman Biomanufacturing, Madison, WI, at 3 mg/mL in PBS (lot no. PPV-pBI-11-FP-001). The DNA plasmid expressing firefly luciferase, pcDNA3-luciferase, was described previously and was prepared with endotoxin-free Qiagen kits (21).

### Detection of luciferase expression

Twenty-four-week-old female C57BL/6 mice were anesthetized with ketamine and injected with pcDNA3-luciferase plasmid at three different locations. The first location was on the back of the mice with a total of 10 µg/50 µL/mouse of pcDNA3-luciferase injected through I.D. injection with a needle-free biojector device from PharmaJet (Golden, CO) customized for use in mice (modified for 0.05-mL delivery) with the C503-32 Tropis Syringe (loaded via a C503-42 Tropis Filling Adapter). This customized needle-free Tropis injects 50 µL instead of the standard 100-µL volume. The second location was on the left hind leg muscle with a total of 10 µg/50 µL/mouse of pcDNA3-luciferase injected through I.M. injection with the customized needle-free biojector. The third location was on the right hind leg muscle with a total of 10 µg/50 µL/mouse of pcDNA3-luciferase injected through I.M. injection with needle. The expression of luciferase was monitored by imaging with IVIS Lumina III In Vivo Imaging System (PerkinElmer, Inc., Waltham, MA) at indicated time.

### Vaccination

Female C57BL/6 mice (11–12 weeks old) were used for the vaccination study. To compare the immunogenicity of pBI-11 between administration at single site (one limb) and multiple sites (all four limbs) of I.M. injection, DNA was diluted from cGMP grade stock with PBS to the concentration of 0.4 and 0.1 µg/µL, respectively. For single-site (one limb) injection, 20 µg/50 µL of pBI-11 DNA was injected into the right rear leg muscle. For multiple-site (four limbs) injection, 5 µg/50 µL of pBI-11 DNA was injected into the muscle of all limbs (total of 20 µg). The mice were boosted twice with the same dose and regimen at 7-day intervals. To compare the immunogenicity of pBI-11 administration through either needle or needle-free device, in naïve or TC-1 tumor-bearing C57BL/6 mice, DNA was diluted from cGMP grade stock with PBS to the concentration of 0.5 µg/µL. For pBI-11 DNA I.D. injection with needle-free device, mice were anesthetized with ketamine (1.35 mg, I.M. injection), and 25 µg/50 µL was injected on each side of the back skin (total of 50 µg). For pBI-11 DNA I.M. injection with either needle or needle-free device, mice were anesthetized with ketamine, and 25 µg/50 µL was injected into the muscle of each rear leg (total of 50 µg). These mice were boosted twice with the same dose and regimen at 1-week intervals.

### Tetramer staining

For tetramer staining, mouse peripheral blood mononuclear cells (PBMCs) were stained with purified anti-mouse CD16/32 first, and then stained with FITC-conjugated anti-mouse CD8a, and PE-conjugated HPV16/E7aa49–57 peptide-loaded H-2D^b^ tetramer at 4°C for 1 hour. After washing, the cells were stained with 7-AAD. The cells were acquired with FACSCalibur flow cytometer and analyzed with CellQuest Pro software (BD Biosciences, Mountain View, CA).

### Intracellular cytokine staining

To detect HPV16 E6/E7 or HPV18 E6-specific CD8^+^ T cell responses by IFN-γ intracellular staining, splenocytes from vaccinated or naïve C57BL/6 mice were prepared and stimulated with either HPV16 E6aa50-57 peptide (5 µg/mL), HPV16 E7aa49-57 peptide, or HPV18 E6aa67-75 peptide (1 µg/mL) in the presence of GolgiPlug (1 µL/mL) at 37°C overnight. The stimulated splenocytes were then washed with PBS containing 0.5% BSA and stained with PE-conjugated anti-mouse CD8a antibody. Cells were fixed and permeabilized according to the manufacturer’s instruction (eBioscience). The cells were further stained for intracellular IFN-γ with FITC-conjugated anti-mouse IFN-γ antibody. Flow cytometry analysis was performed using FACSCalibur flow cytometer with CELLQuest software (BD Biosciences).

### *In vivo* tumor treatment experiment

To compare the therapeutic effect of pBI-11 DNA vaccine administered through either needle or needle-free device, 11- to 12-week-old female C57BL/6 mice (six to eight per group) were implanted with 2 × 10^5^ of TC-1 tumor cells subcutaneously. The tumor-bearing mice were divided into four groups. The first group of mice did not receive any vaccination (untreated). For the remaining groups, pBI-11 vaccination was initiated on day 3 after TC-1 tumor cell challenge while mice were anesthetized with ketamine. One group of TC-1 tumor-bearing mice were vaccinated with 25 µg/50 µL of pBI-11 DNA by injection into the muscle of each rear leg (total of 50 µg) with a needle (pBI-11 [I.M. with needle]). Another group of mice was vaccinated with 25 µg/50 µL of pBI-11 DNA by injection into the muscle of each rear leg (total of 50 µg) with the customized needle-free biojector (pBI-11 [I.M. with needle-free]). The last group of mice was injected intradermally with 25 µg/50 µL of pBI-11 DNA on each side (total of 50 µg) with customized needle-free biojector (pBI-11 [I.D. with needle-free]) into the back skin pulled into a fold and the device oriented perpendicular to the skin and away from the muscle. These mice were boosted twice with the same dose and regimen using the schedule as described (see Fig. 4A). The growth of tumor was monitored twice a week by palpation and digital caliper measurement. Tumor volume was calculated using the formula [largest diameter × (perpendicular diameter)2] × 3.14/6. To record the survival of the tumor-bearing mice, either natural death or a tumor diameter greater than 2 cm leading to death was counted as death.

### Characterization of T-cell phenotype, regulatory T cells, and myeloid-derived suppressor cells in TC-1 tumor-bearing mice after vaccination

To analyze the T-cell phenotype, regulatory T cells and myeloid-derived suppressor cells (MDSCs) in PBMC of TC-1 tumor-bearing C57BL/6 mice after pBI-11 DNA vaccination, with either needle or needle-free device, blood was collected 32 days after TC-1 tumor cell challenge and stained with BV780-conjugated anti-mouse CD3, FITC-conjugated anti-CD4, BV421-conjugated anti-mouse CD8a antibody, APC-A700-conjugated anti-mouse CD44, and PE-conjugated anti-mouse CD62L antibodies. After permeabilization/fixation, the cells were further stained with PE-Cy5-conjugated anti-mouse Foxp3 antibody. The cells were acquired with CytoFlex S flow cytometer (Beckman Coulter, Brea, CA) and analyzed with FlowJo software from BD Biosciences (Franklin Lakes, NJ). To analyze MDSC, PBMCs from TC-1 tumor-bearing mice were stained with FITC-conjugated anti-mouse Gr-1 (Ly-6G) and PE-conjugated anti-mouse CD11b antibodies. The cells were acquired with FACSCalibur flow cytometer and data were analyzed with CellQuest Pro software (BD Biosciences).

### Impact of vaccination on behavior and physiological status of mice

To assess the impact of pBI-11 DNA vaccination through either needle or needle-free device of 11- to 12-week-old naïve female C57BL/6 mice (five mice per group), their DNA was diluted from cGMP grade stock (lot no. PPV-pBI11-FP-001; Waisman Biomanufacturing) with PBS to the concentration of 0.5 µg/µL. For pBI-11 DNA I.D. injection with customized needle-free biojector, mice were anesthetized with ketamine, and 25 µg/50 µL was injected on each side of the back skin (total of 50 µg). For pBI-11 DNA I.M. injection with either needle or customized needle-free biojector, mice were anesthetized with ketamine, and 25 µg/50 µL was injected into the muscle of each rear leg (total of 50 µg). These mice were boosted twice with the same dose and regimen with a 7-day interval. The health of mice was monitored by the measurement of behaviors, body weight, and injection site irritation throughout the duration of vaccination and up to 1 week post-final vaccination per the JHU Animal Pathobiology and Phenotyping manual. Seven days after the last vaccination, complete blood counting was performed at the JHU Animal Pathobiology and Phenotyping Core. A comprehensive chemistry analysis was performed by IDEXX BioAnalytics (West Sacramento, CA). In addition, necropsy was performed 1 week after the last vaccination (day 21); key organ weights were measured, and histology was examined by a board-certified pathologist. Approximately one-half of each spleen was used for histological analysis, and the remainder was used to prepare single splenocytes and was stimulated with HPV16 E6, HPV16 E7, or HPV18 E6 peptides followed by IFN-γ intracellular staining.

### Statistical analysis

Comparisons between each vaccine delivery approach in expression kinetics of luciferase over time were conducted via paired *t*-test (in log 10 scale of intensity) and with multiplicity adjustment for *P* values via Holm approach (22). For continuous measurements such as the percentage of the antigen-specific CD8+ T cells or organ weights, the group differences were assessed using Kruskal-Wallis test if three or more groups were compared or Wilcoxon rank-sum test if two groups were compared. In cases where significant differences were observed among three or more groups, post hoc analyses were conducted using the Wilcoxon rank-sum test for pairwise comparisons with multiplicity adjustment using the Holm approach. Tumor growth and weight changes over time by untreated and different vaccine delivery approaches were evaluated via linear mixed effects models (LMMs) (23), where an exponential spatial correlation structure was assumed for the longitudinal measures within individual mouse. The tumor volume was log 10 transformed in the analysis to reduce distribution skewness and to approximate linearity in the growing trend over time. The overall differences in the rate of tumor growth or rate of weight changes among untreated and vaccine delivery approaches were evaluated by testing the interaction term of the treatment group and the time from the LMM. The *P* values of pairwise comparisons for tumor growth rate were obtained from the same LMM model and adjusted for multiplicity using the Holm approach. Survival analysis was conducted via Kaplan-Meier method, and overall difference among untreated and three vaccine delivery approaches was examined using the log-rank test, followed by pairwise comparisons of survival time between each other untreated or vaccination groups using log-rank test with multiplicity adjustment using the Holm approach.

The significance threshold was set at a two-sided *P* value of <0.05. Statistical analyses were performed using Prism software (version 9.5.1) or in R (version 4.2.2) environment (R Foundation for Statistical Computing, Vienna, Austria).

## RESULTS

### pBI-11 DNA vaccination delivered intramuscularly to four limbs generated greater percentages of HPV-specific CD8+ T cells compared to delivery to one limb

**FIG 1:**
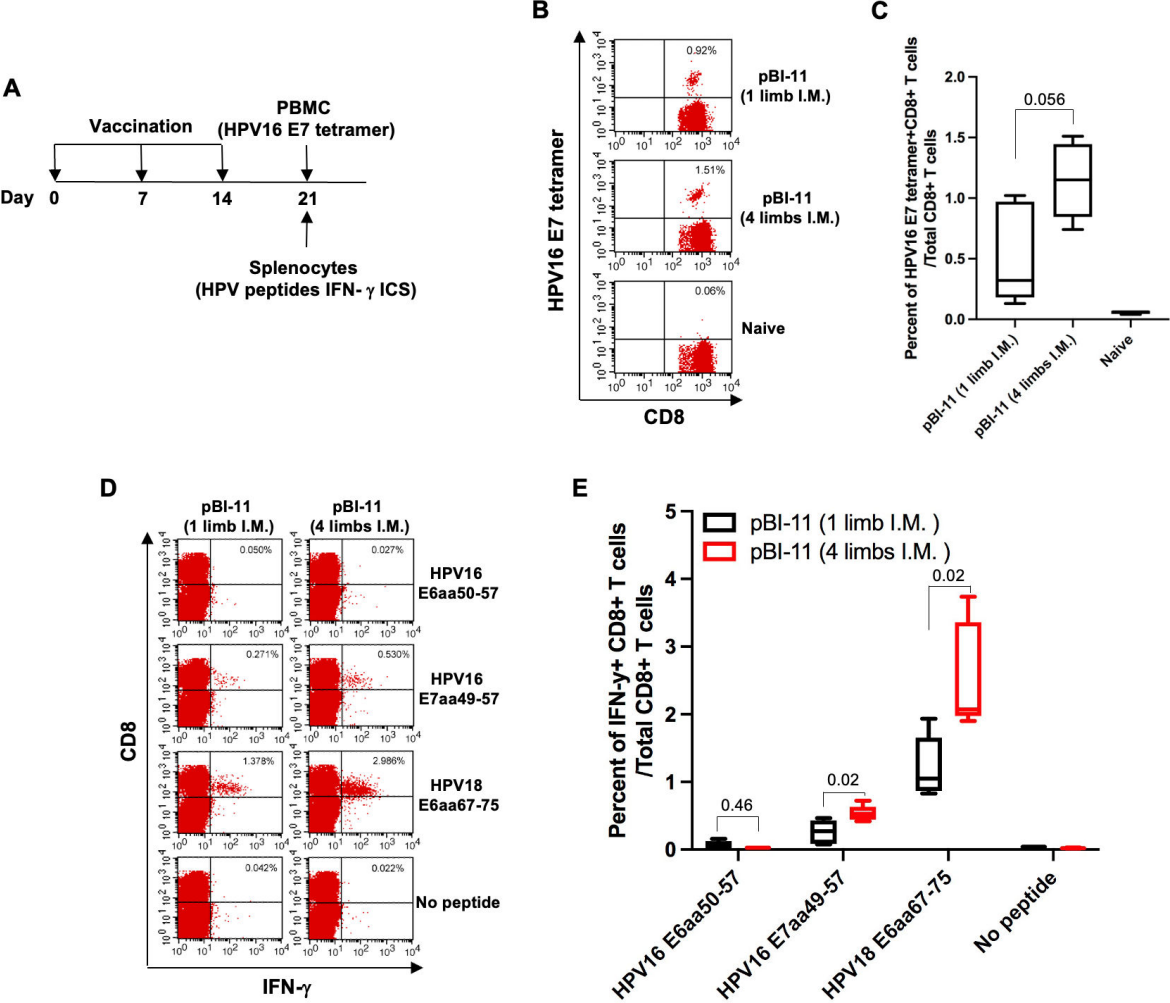
Comparison of HPV16 E6E7 and HPV18 E6-specific CD8+ T-cell responses generated by pBI-11 DNA vaccine between single-site and multiple-site injections. (A) Schema of the vaccinations. Female C57BL/6 mice (11–12 weeks old, five mice/group) were vaccinated with total of 20 µg of pBI-11 DNA (20 µg/50 µL, single limb, or 5 µg/50 µL, four limbs) through I.M. injection on day 0. The mice were boosted twice with the same regimen in a 7-day interval. On day 21, peripheral blood was collected from the vaccinated or naïve mice. PBMCs were prepared by lysing red blood cells and stained with FITC-conjugated anti-mouse CD8a and phycoerythrin (PE)-conjugated, HPV16 E7aa49-57 peptide-loaded H-2D^b^ tetramer. 7-AAD was added to the cells prior to acquisition. Alternatively, splenocytes were prepared from pBI-11 DNA vaccinated mice and stimulated with the following peptides at the presence of GolgiPlug overnight at 37°C: HPV16 E6aa50-57 (5 µg/mL), HPV16 E7aa49-57 (1 µg/mL), and HPV18E6aa67-75 (1 µg/mL). The cells were stained with PE-conjugated anti-mouse CD8a antibody. After permeabilization and fixation, the cells were further stained with FITC-conjugated anti-mouse IFN-γ antibody. The cells were acquired with FACSCalibur flow cytometer and data were analyzed with CellQuest pro software. (B) Representative flow cytometry image of HPV16 E7 tetramer staining. (C) Summary of HPV16 E7 tetramer-specific CD8+ T-cell flow cytometry analysis data. (D) Representative flow cytometry image of HPV16 E6/E7 and HPV18 E6 peptide-specific CD8+ T cells. (E) Summary of HPV16 E6/E7 and HPV18 E6 peptide-specific CD8+ T cells analyzed with flow cytometry.

To compare the HPV-specific CD8+ T cells delivered as a single dose to one limb or as quarter doses delivered in all four limbs, we vaccinated 11- to 12-week-old female C57BL/6 mice with a total of 20 µg of pBI-11 DNA (20 µg/50 µL, single limb, or 5 µg/50 µL, four limbs) through I.M. injection with a syringe and needle. Peripheral blood was collected, and splenocytes were harvested on day 21 for flow cytometry analysis (see Fig. 1A).

Since pBI-11 DNA vaccine targets both HPV16 and 18 E6/E7, we first analyzed HPV16 E7aa49-57 peptide-specific CD8+ T cells using HPV16 E7 tetramer in the peripheral blood. As shown in Fig. 1B and C, our results revealed that mice vaccinated in four limbs trended towards a greater CD8+ HPV16 E7 tetramer+ T-cell response in peripheral blood than that of mice vaccinated in one limb (median of 1.15% vs 0.32%, *P* = 0.056). We then analyzed HPV16 E6aa50-57, HPV16 E7aa49-57, and HPV18 E6aa67-75 peptide-specific CD8+ T cells in spleen from pBI-11 DNA vaccinated mice. As shown in Fig. 1D and E, mice vaccinated in four limbs had significantly greater HPV16 E7aa49-57 (*P* = 0.02) and HPV18 E6aa67-75-specific (*P* = 0.02) CD8+ T-cell responses compared to mice vaccinated in one limb, while HPV16 E6aa50-57-specific CD8+ T-cell responses were weak but comparable between the two methods.

Taken together, our results indicate that I.M. injection of pBI-11 DNA vaccination in four limbs generated better HPV-specific CD8+ T-cell responses than vaccination in only one limb, consistent with prior studies (12). Since two-site vaccination with 50% of the dose led to better HPV-specific T-cell responses in patients (13), we delivered customized needle-free DNA vaccination at two locations at each time point in the subsequent experiments.

### Naked plasmid DNA delivered through customized needle-free biojector leads to enhanced marker gene expression compared to injection with conventional needle device

To compare the level and the kinetic expression of encoded genes by DNA vector delivered through the customized needle-free biojector or conventional needle and syringe, we used pcDNA3-luciferase plasmid. The pcDNA3-luciferase plasmid was administered to 24-week-old C57BL/6 mice via I.D. injection using a customized needle-free biojector, I.M. injection using a customized needle-free biojector, or I.M. injection using a conventional needle device. The expression of luciferase in injected mice were followed up over time using non-invasive IVIS Lumina III In Vivo Imaging System after injection of luciferin substrate.

**FIG 2:**
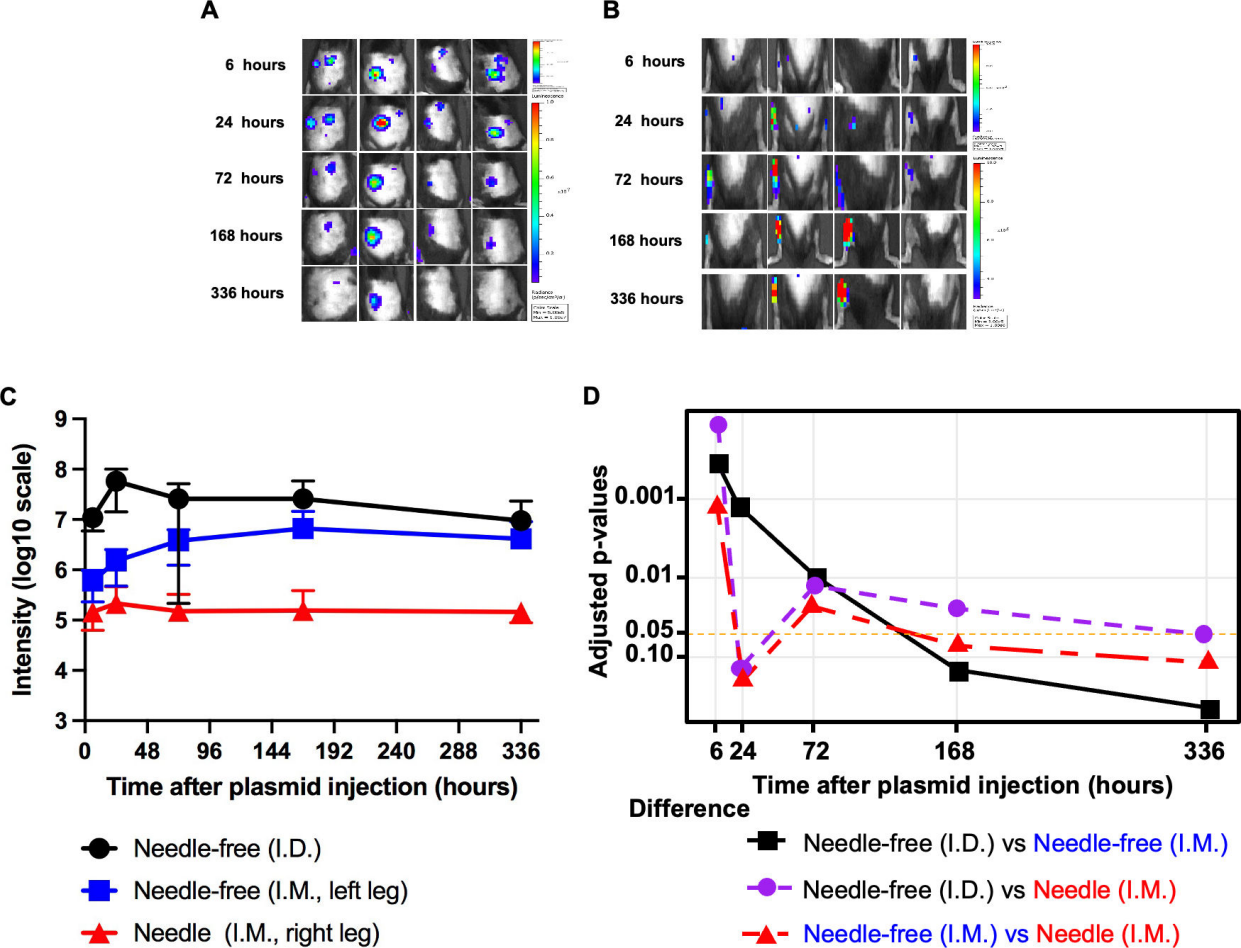
Comparison of luciferase expression after pcDNA3-luciferase needle or needle-free device injection. Twenty-four-week-old female C57BL/6 mice (four mice/group) were anesthetized with ketamine. These mice were injected with pcDNA3-luciferase plasmid at three different locations. The first location was on the back of the mice with a total of 10 µg/50 µL/mouse of pcDNA3-luciferase, which was injected intradermally with a customized needle-free biojector from PharmaJet. The second location was on the left hind leg muscle with a total of 10 µg/50 µL/mouse of pcDNA3-luciferase, which was injected intramuscularly with customized needle-free biojector. The third location was on the right hind leg muscle with a total of 10 µg/50 µL/mouse of pcDNA3-luciferase, which was injected through an I.M. injection with a conventional needle. The expression of luciferase was monitored by imaging with IVIS lumina III system. A. bioluminescence image of luciferase expression after pcDNA3-luciferase plasmid I.D. injection with needle-free biojector. (B) Bioluminescence image of luciferase expression after pcDNA3-luciferase plasmid I.M. injection with either customized needle-free biojector or needle. (C) Summary of luciferase expression over time by vaccine delivery approaches. (D) Adjusted *P* values using Holm method for testing differences in luciferase expression between each of the injection delivery approaches over time.

The expression of luciferase demonstrated different kinetics in mice injected using a customized needle-free biojector, either though I.D., I.M., or I.M. using conventional needle device. The expression of luciferase in mice injected with a customized needle-free biojector demonstrated high expression at 6 hours after injection and peaked at 24 hours and gradually decreased thereafter (Fig. 2A and C). In comparison, the expression of luciferase appeared to peak at 168 hours after the injection in mice injected I.M. using a customized needle-free biojector (Fig. 2B and C). Among the three injection approaches, I.D. injection with a customized needle-free biojector demonstrated the strongest expression of luciferase (Fig. 2C). As shown in Fig. 2D, the expression level utilized by I.D. injection is significantly higher than I.M. injection with conventional needle device at 6, 72, and 168 hours after injection. When compared to I.M. injection with a customized needle-free biojector, the expression of luciferase via I.D. injection is significantly higher at 6, 24, and 72 hours. Furthermore, the expression of luciferase of mice injected I.M. with a customized needle-free biojector is significantly higher than the mice injected I.M. with conventional needle device at 6 and 72 hours (Fig. 2D). We monitored the luciferase bioluminescence from all three injection approaches up to 336 hours; it appeared that both I.D. or I.M. injection using a customized needle-free biojector showed higher expression of luciferase than using I.M. injection with conventional needle device across over the followed-up time frame (Fig. 2D).

Taken together, our data indicate that DNA expressing vector delivered either I.D. or I.M. using the customized needle-free biojector produced different expression kinetics, but both enhanced gene expression compared to injection with a conventional needle device.

### pBI-11 DNA vaccination administered intradermally with customized needle-free biojector generated increased the number of HPV-specific CD8+ T-cell responses compared to needle-based intramuscular injection

**FIG 3:**
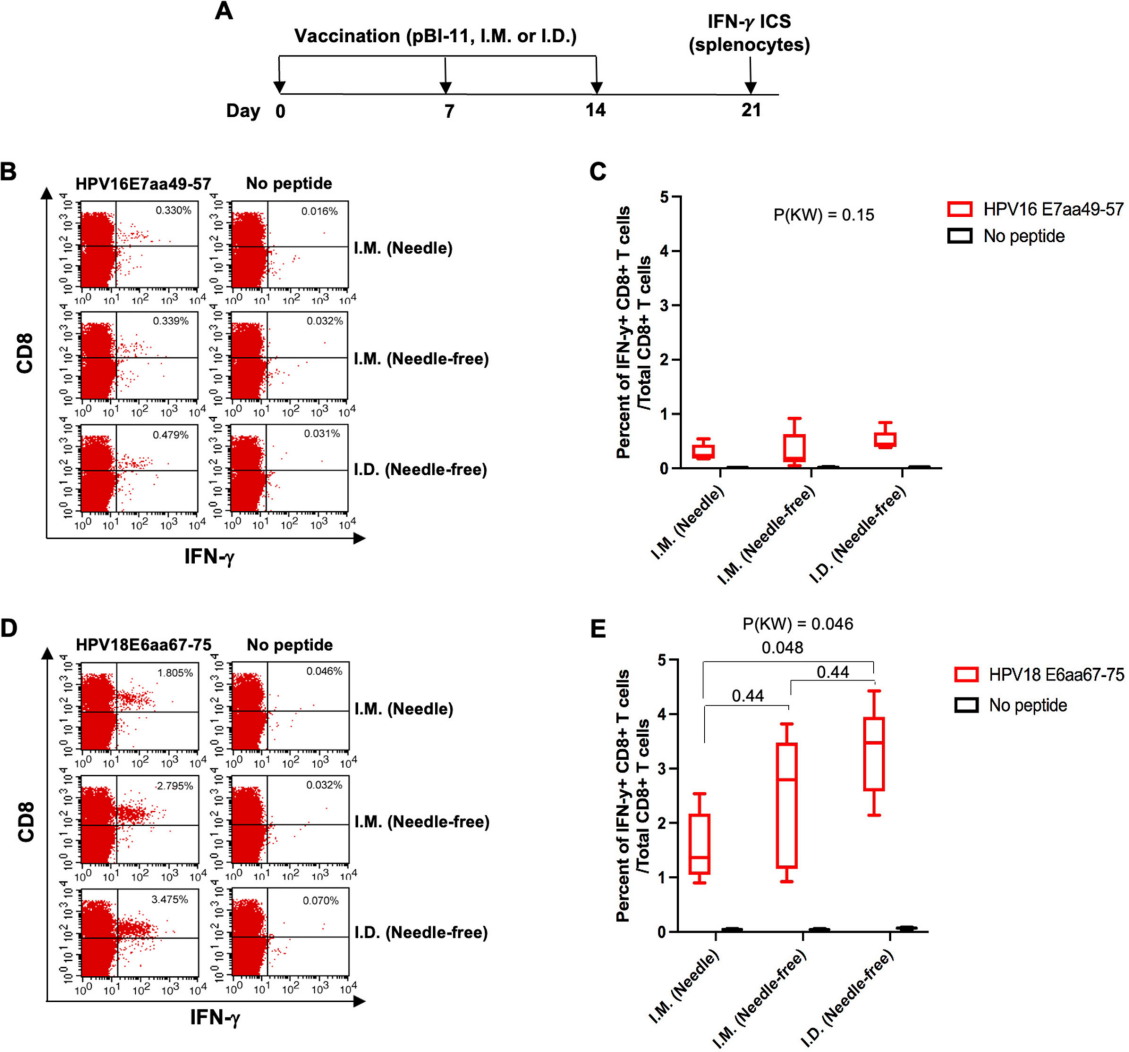
Comparison of HPV-specific CD8+ T-cell responses after pBI-11 DNA vaccination with either needle or needle-free device. A. schema of the experiment. One group of 11- to 12-week-old female C57BL/6 mice (five mice/group) were vaccinated with a total of 50 µg of pBI-11 DNA (25 µg/50 µL/mouse per injection with two separate injections on each hind leg) through I.M. injection with needle. A second group of 11- to 12-week-old female C57BL/6 mice (5 mice/group) were vaccinated with a total of 50 µg of pBI-11 DNA (25 µg/50 µL/mouse per injection with two separate injections on each hind leg) through I.M. injection with customized needle-free biojector under anesthesia with ketamine. A third group of 11- to 12-week-old female C57BL/6 mice (five mice/group) were vaccinated with a total of 50 µg of pBI-11 DNA (25 µg/50 µL/mouse per injection with two separate injections) through I.D. injection on the back with customized needle-free biojector under anesthesia with ketamine. These mice were boosted twice with the same regimen at a 1-week interval. Seven days after the last vaccination, splenocytes were prepared from these vaccinated mice and stimulated with either HPV16 E7aa49-57 peptide (1 µg/mL), or HPV18 E6aa67-75 peptide (1 µg/mL) in the presence of GolgiPlug (1 µL/mL overnight. The cells were stained with PE-conjugated anti-mouse CD8a (clone 53.6.7) at 4°C for 30 min. After washing, the cells were permeabilized and fixed with perm/fix buffer (from eBioscience) at 4°C for 30 min. After washing, the cells were further stained with FITC-conjugated anti-mouse IFN-γ (clone XMG1.2) at 4°C for 45 min. The cells were resuspended in PBS + 0.5% BSA after washing. The cells were acquired with FACSCalibur flow cytometer, and data were analyzed with CellQuest Pro software. (B) Representative flow cytometry images of HPV16 E7aa49-57 peptide-specific CD8+ T cells detected by IFN-γ intracellular staining assay. (C) Summary of HPV16 E7aa49-57 peptide-specific CD8+ T cells detected by IFN-γ intracellular staining assay after pBI-11 DNA vaccination. Data shown as boxplots displaying the median in the centre line with interquartile range at top and bottom of the box and the 10th/90th percentile at the whiskers. (D) Representative flow cytometry images of HPV18 E6aa67-75 peptide-specific CD8+ T cells detected by IFN-γ intracellular staining assay. (E) Summary of HPV18 E6aa67-75 peptide-specific CD8+ T cells detected by IFN-γ intracellular staining assay after pBI-11 DNA vaccination. All other pair-wise *P* values were from Wilcoxon test with multiplicity adjustment using Holm method. *P*(KW), *P* value based on Kruskal-Wallis test for global difference.

To evaluate the HPV-specific CD8+ T cell-mediated immune responses in C57BL/6 mice vaccinated with pBI-11 DNA using a conventional needle and syringe or a customized needle-free biojector, we performed intracellular cytokine stains followed by flow cytometry analysis on the splenocytes derived from vaccinated mice. Mice were vaccinated with split dosing three times at 1-week intervals, and their splenocytes were harvested 7 days after the final vaccination, as shown in the schema in Fig. 3A.

Our flow cytometry results revealed that mice vaccinated I.D. with the customized needle-free biojector showed increased numbers of HPV16 E7aa49-57 peptide-specific CD8+ T-cell responses (median 0.45%) compared to mice vaccinated intramuscularly with a customized needle-free biojector (median 0.18%) or a conventional needle device (median 0.23%) (Fig. 3B and C). However, these results were not statistically significant. Interestingly, for the HPV18 E6aa67-75 peptide-specific CD8+ T cell-mediated immune responses (the dominant epitope), mice vaccinated I.D. with the customized needle-free biojector demonstrated significantly (*P* = 0.048) increased numbers of HPV18 E6aa67-75 peptide-specific CD8+ T-cell responses (median 3.48%) compared to mice vaccinated intramuscularly with a syringe and needle (median 1.37%) (Fig. 3D and E). Furthermore, pBI-11 DNA vaccination delivered I.M. by the customized needle-free biojector generated numerically better CD8+ T cell-mediated immune responses (median 2.80%) compared to I.M. delivery by the conventional needle injection (median 1.37%), although these differences were not statistically significant.

These data indicate that mice vaccinated I.D. with a customized needle-free biojector is the best way to generate HPV antigen-specific CD8+ T cells compared to other routes of administration we tested.

### C57BL/6 mice vaccinated I.D. with pBI-11 DNA using customized needle-free biojector demonstrated enhanced therapeutic anti-tumor immunity relative to vaccination using conventional needle injection

**FIG 4:**
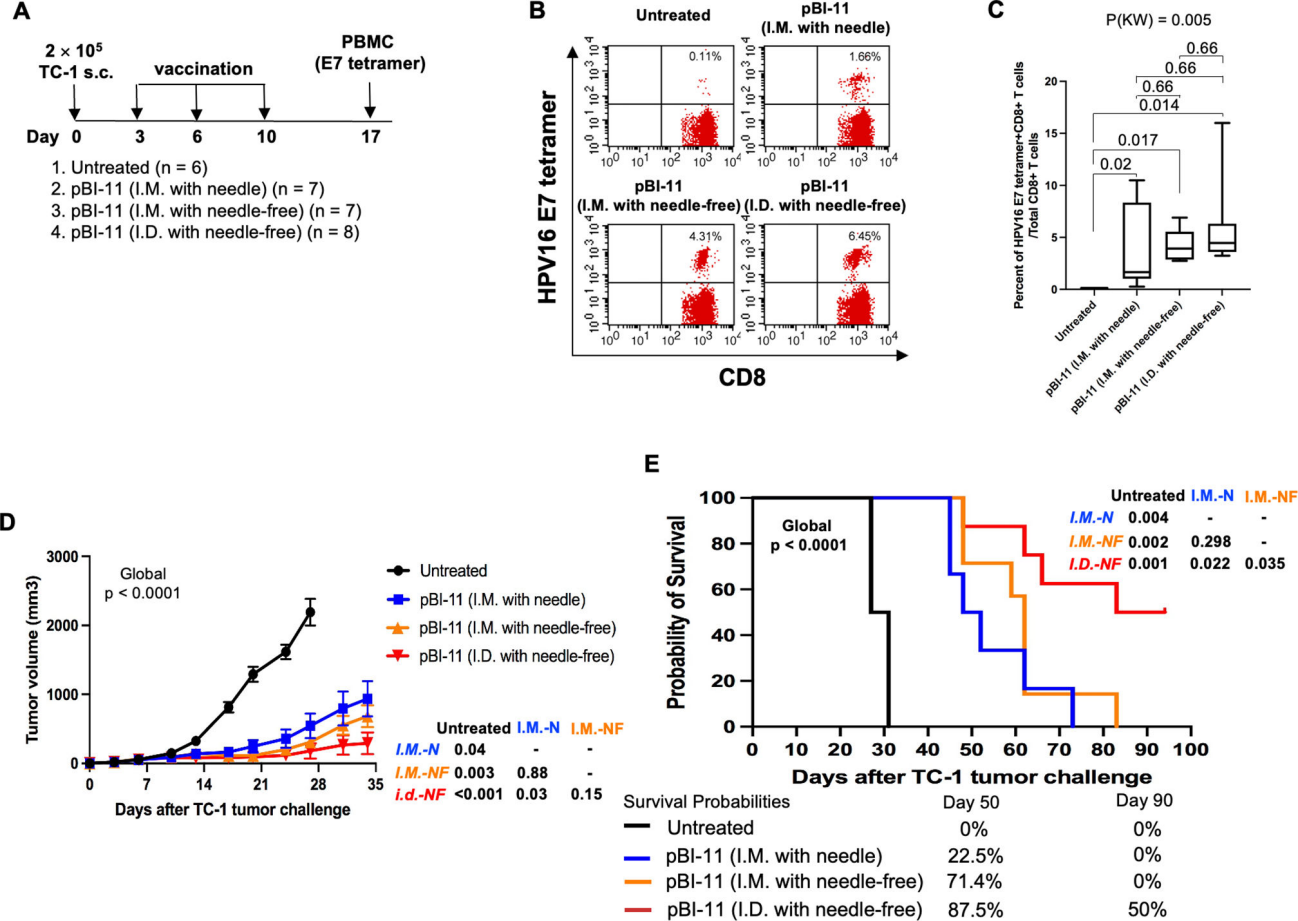
Comparison of anti-tumor immunity in TC-1 tumor-bearing B6 mice after pBI-11 DNA vaccination using needle or needle-free device. Experimental procedure: female C57BL/6 mice (11–12 weeks old, six to eight mice/group) were injected with 2 × 10^5^ of TC-1 cells subcutaneously on day 0. On day 3, the mice were divided into four groups. The first group was used as the untreated control. The mice of the second group were vaccinated with a total of 50 µg of pBI-11 DNA (25 µg/50 µL/mouse per injection with two separate injections) through I.M. injection with needle. The mice were further boosted with the same regimen at the indicated interval. The mice of the third group were vaccinated with a total of 50 µg of pBI-11 DNA (25 µg/50 µL/mouse per injection with two separate injections) through I.M. injection with customized needle-free biojector under anesthesia with ketamine. The mice were boosted twice with the same regimen at the indicated interval. The mice of the fourth group were vaccinated with a total of 50 µg of pBI-11 DNA (25 µg/50 µL/mouse per injection with two separate injections) through I.D. injection on the back using customized needle-free biojector under anesthesia with ketamine. The mice were boosted twice with the same regimen at the indicated interval. The growth of the tumor was monitored twice a week by palpation and digital caliper measurement. Tumor volume was calculated using the formula [largest diameter × (perpendicular diameter)^2^] × 3.14/6. To record the survival of the tumor-bearing mice, either natural death or a tumor diameter greater than 2 cm leading to death was counted as death. (A) Schema of the experiment. (B) Representative flow cytometry image of HPV16 E7-specific CD8+ T cells in peripheral blood using HPV16 E7 tetramer staining. (C) Summary of HPV16 E7-specific CD8+ T cells in peripheral blood detected by HPV16 E7 tetramer staining. (D) Summary of the TC-1 tumor volume of the mice. (E) Kaplan-Meier analysis of the survival of TC-1 tumor-bearing mice. The survival probabilities for days 50 and 90 are shown under panel E *.* All other pair-wise *P* values were from Wilcoxon test with multiplicity adjustment using the Holm method. I.D.-NF-pBI-11, I.D. with needle-free; I.M.-N-pBI-11, I.M. with needle; I.M.-NF-pBI-11, I.M. with needle-free. *P*(KW), *P* value based on Kruskal-Wallis test for global difference.

We evaluated the HPV antigen-specific CD8+ T cell-mediated immune responses as well as therapeutic anti-tumor effects in TC-1 tumor-bearing female C57BL/6 mice treated with pBI-11 DNA vaccination using three different delivery methods: (i) I.M. with a conventional needle and syringe, (ii) I.M. with a customized needle-free biojector, or (iii) I.D. with a customized needle-free biojector. The experiment was performed as depicted in Fig. 4A, and the comparisons were conducted among different vaccine delivery approaches with post hoc comparisons between the vaccine delivery approaches. PBMCs were analyzed with HPV16 E7 tetramer 7 days after the final vaccination. Tumor growth was monitored by palpation and digital caliper method twice a week.

As shown in Fig. 4B and C, our results revealed that pBI-11 DNA vaccination by the customized needle-free biojector (intradermally or intramuscularly) produced comparable levels of circulating HPV antigen-specific CD8+ T cells relative to conventional needle injection of TC-1 tumor-bearing mice. pBI-11 DNA delivered through all these different delivery methods generated greater HPV16 E7-specific CD8+ T-cell immune responses compared to the untreated control group.

The examination of tumor volume was conducted until day 27, when all mice were still alive. Our results showed TC-1-bearing mice treated with pBI-11 DNA delivered by each of the three methods exhibited significantly reduced tumor volume compared to the untreated group (adjusted *P* < 0.05) (Fig. 4D). In addition, tumor volume was significantly slower in mice vaccinated by I.D. injection with a customized needle-free biojector than mice vaccinated by I.M. injection with conventional needle (adjusted *P* = 0.02). However, no significant difference in tumor volume was observed when mice vaccinated using I.M. approach with conventional needle were compared to a customized needle-free biojector (adjusted *P* = 0.46). Vital status of mice was recorded up to 94 days after tumor challenge. All untreated mice were dead by 31 days after TC-1 tumor challenge. Vaccination of mice with pBI-11 DNA by all three delivery methods significantly extended survival compared with untreated mice (Fig. 4E, all adjusted *P* < 0.005). When comparing survival differences by vaccine delivery approaches, mice vaccinated by I.D. injection with a customized needle-free biojector yielded superior survival to both I.M. injection with conventional needle device (adjusted *P* = 0.02) and I.M. injection with a customized needle-free biojector (adjusted *P* = 0.04); all mice vaccinated by I.M. routes died within 90 days after tumor challenge, and the survival probability at 90 days was 50% (95% CI 25.0–99.9%) in mice vaccinated by I.D. with a customized needle-free biojector. There was no statistically significant difference in survival when comparing mice treated with an I.M injection with a customized needle-free biojector to I.M. injection with conventional needle and syringe (adjusted *P* = 0.30).

Overall, these data indicate that mice vaccinated with pBI-11 DNA using the customized needle-free biojector intramuscularly generated comparable tumor treatment results to vaccination with a conventional needle intramuscular injection. In contrast, I.D. vaccination with pBI-11 DNA in TC-1 tumor-bearing mice using the customized needle-free biojector generated significantly better anti-tumor effect and longer survival compared to vaccination with conventional needle I.M. injection.

### pBI-11 DNA vaccination regardless of vaccine delivery approaches significantly reduced the number of myeloid-derived suppressor cells in PBMCs of TC-1 tumor-bearing mice

**Fig 5:**
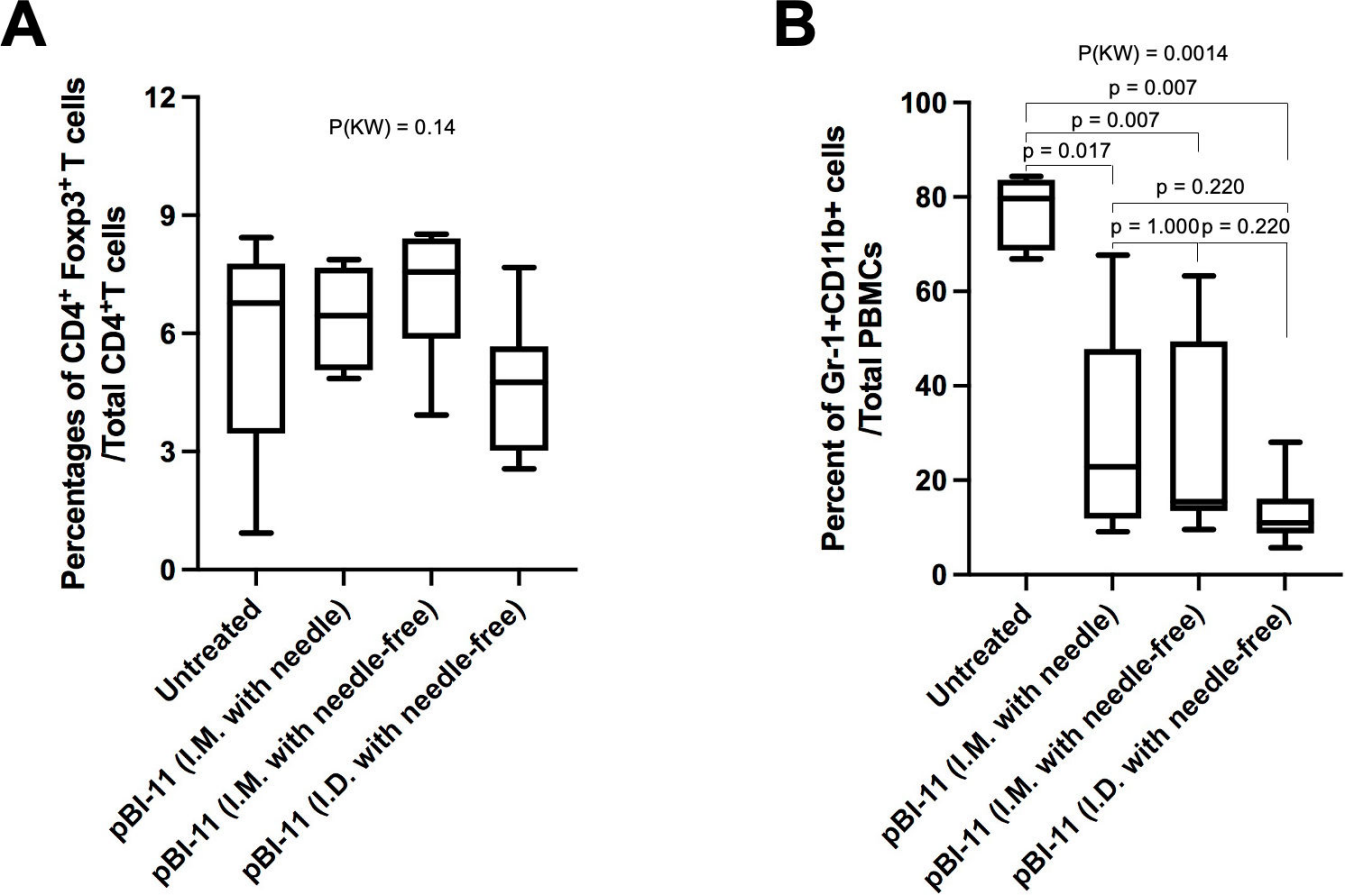
Comparison of regulatory T cells and MDSC in PBMC from TC-1 tumor-bearing C57BL/6 mice. Female TC-1 tumor-bearing C57BL/6 mice were either untreated or treated as described in Fig. 4A. On day 32 after TC-1 tumor cell challenge, PBMCs were collected from the mice, and the frequencies of regulatory T cells (**A**) and MDSC (**B**) were analyzed using flow cytometry analysis. All other pair-wise *P* values were from Wilcoxon test with multiplicity adjustment using Holm method. MDSC, myeloid-derived suppressor cell; *P*(KW), *P* value based on Kruskal-Wallis test for global difference.

We also assessed regulatory T cell and MDSC numbers in peripheral blood of the TC-1 tumor-bearing mice after vaccination with pBI-11 DNA I.D. with a customized needle-free biojector, I.M. with a customized needle-free biojector, or I.M. with a conventional needle device as compared to unvaccinated animals. No significant differences in percentage of regulatory T cells was observed in the PBMC harvested on day 32 after the TC-1 tumor challenge (22 days after the last pBI-11 DNA vaccination) between untreated mice and vaccinated mice, regardless of the vaccination delivery method employed (Fig. 5A). However, we observed a significantly lower percentage of MDSC in mice vaccinated with pBI-11 using any of the three delivery methods, when compared to unvaccinated mice (Fig. 5B). Furthermore, MDSCs in PBMCs from tumor-bearing mice treated with pBI-11 delivered intradermally with a customized needle-free biojector trended lower than I.M. delivery with or without a customized needle-free biojector, although this was not statistically significant.

Taken together, these data indicate tumor-bearing mice treated with pBI-11 delivered via each of the three different methods resulted in significantly lower MDSCs, but not regulatory T cells, in PBMCs compared to untreated mice.

We also characterized the overall CD4+ T cells in PBMCs harvested from TC-1 tumor-bearing mice on day 32 after the TC-1 tumor cell challenge in groups treated with pBI-11 DNA delivered through different routes of administration versus untreated. We found that pBI-11 DNA vaccination was associated with greater CD3+ CD4+ T cell and CD44^+^CD62L^−^ effector CD4 T-cell levels compared to untreated mice, while I.D. injections with a customized needle-free biojector resulted in a greater percentage of CD3+ CD4+ T cells compared to untreated mice and mice vaccinated intramuscularly with a conventional needle device (see Fig. S1A). Our data also showed mice vaccinated with pBI-11, using any of the three methods, had significantly greater percentages of CD44^+^CD62L^−^ effector CD4 T cells compared to untreated mice. However, the CD44^+^CD62L^−^ effector CD4 T cells were comparable among the three vaccinated groups of mice, and no statistical significance was demonstrated (see Fig. S1B).

We also characterized the overall CD8+ T cells in PBMCs derived from TC-1 tumor-bearing mice treated with pBI-11 DNA delivered through different routes of administration. We found that pBI-11 DNA vaccination intradermally with a customized needle-free biojector resulted in the highest percentage of CD3^+^ CD8^+^ T cells among all of the treatment groups (see Fig. S2).

### pBI-11 DNA vaccination delivered by PharmaJet customized needle-free biojector is well tolerated

Previously, we have demonstrated that pBI-11 DNA vaccination delivered through I.M. injection by needle is safe and well tolerated in mice (11). In addition, the clinical-grade pBI-11 DNA vaccination is being tested in hrHPV+ patients with advanced head and neck cancers (NCT05799144), and its precursor pBI-1 was well tolerated in HPV16-infected patients with cervical low-grade or high-grade disease or HPV16+ head and neck cancer. However, it is unclear whether the pBI-11 DNA vaccine is well tolerated when administered intradermally or intramuscularly using the customized needle-free biojector. To investigate this, we conducted an experiment with 11- to 12-week-old female C57BL/6 mice (five mice/group), which were divided into three groups. The mice of the first group (naïve) were left unvaccinated. The mice of the second group were vaccinated with a total of 50 µg of pBI-11 DNA (25 µg/50 µL/mouse per injection with two separate injections) through I.D. injection on the back using the customized needle-free biojector. The mice of the third group were vaccinated with a total of 50 µg of pBI-11 DNA (25 µg/50 µL/mouse per injection with two separate injections) on each hind leg through I.M. injection using the customized needle-free biojector. The mice were boosted twice with the same dose and regimen at a 1-week interval.

Throughout the duration of the vaccination, the health of the mice was closely monitored via observation of their body weight, behaviors, and injection site. All vaccinated mice appeared healthy for the entire duration of the experiment, and demonstrated typical behavioral phenotypes for all assessments throughout (Table S1). On the day of vaccination, the injection sites of vaccinated mice were observed at 2 and 24 hours post-vaccination, revealing no eschar (Table S2) or edema formation (Table S3) for mice in any of the vaccination groups at any time point. Moreover, the body weight of mice in all vaccination groups remained consistent and comparable throughout the experimental duration (Fig. S3). We also compared HPV antigen-specific CD8+ T cell-mediated immune responses elicited by pBI-11 DNA administered through I.M. and I.D. via customized needle-free biojector. Our results demonstrated that pBI-11 DNA vaccination administered intradermally through a customized needle-free biojector showed slightly greater HPV antigen-specific CD8+ T-cell immune responses compared to vaccination administered intramuscularly through a customized needle-free biojector. However, there was no statistical significance between these two vaccination groups (Fig. S4). These results are consistent with the data shown in Fig. 3 (see Fig. 3).

One week following the final vaccination, we conducted necropsy and performed key organ weight measurements, complete blood count, clinical chemistry analysis, and histological studies. The mean organ weights were comparable in all vaccination groups (Fig. S5). Complete blood count measurements were all unremarkable and similar in mice of all vaccination groups (Table S4), and biochemistry readouts showed non-significant and comparable results in mice of all vaccination groups (Table S5). Histological examination of key organs also revealed no significant findings, with results within normal limits for mice of all vaccination groups (Table S6). Taken together, these findings suggest that behavioral and physiological status of mice from the naïve group, I.D. via customized needle-free biojector group, and I.M. via customized needle-free biojector group showed similar results, indicating the administration of pBI-11 vaccination using the customized needle-free biojector through I.D. or I.M. injection is safe and well tolerated in mice.

## DISCUSSION

In this study, we observed that DNA expressing vector delivered I.M. through a customized needle-free biojector leads to enhanced gene expression but comparable therapeutic anti-tumor immunity and survival relative to vaccination using conventional needle injection. Notably, pBI-11 DNA vaccination administered I.D. with a customized needle-free biojector generated better anti-tumor responses compared to conventional I.M. injection and longer survival than either conventional I.M. injection or I.M. delivery via customized needle-free biojector. Nevertheless, TC-1 tumor-bearing mice treated with pBI-11 DNA vaccination delivered through different delivery methods all generated a significantly lower number of MDSCs in PBMCs compared to untreated mice. Overall, we have shown that the customized needle-free biojector is safe and well tolerated. Furthermore, given data showing greater acceptability (23) and its use to deliver a licensed DNA vaccine, Tropis is potentially more desirable in delivering pBI-11 DNA vaccine than conventional syringe and needle injection. The transduction of muscle after injection of plasmid DNA appears more efficient in mice than in patients, and because of this species difference, comparison of the Stratis and Tropis devices is warranted.

We observed greater antigen-specific T-cell responses when vaccination was delivered to four vaccination sites instead of one vaccination site (see Fig. 1). This difference in antigen-specific T-cell responses likely is because single-site vaccination delivers DNA vaccine to antigen-presenting cells near the location of injection and the corresponding lymph node, while multiple-location vaccination will deliver the DNA vaccine to different draining lymph nodes, increasing the likelihood of engaging a broader range of antigen-presenting cells and enhancing the antigen-specific T-cell responses (24). Each site of vaccination has its unique microenvironment wherein distinct types of antigen-presenting cells present and process antigens, so targeting multiple vaccination sites may increase the chances of more effective antigen-presenting cells and the subsequent expansion of antigen-specific T cells (25). A preclinical study investigating single-site and multiple-site I.D. DNA vaccination in mice found multiple-site injection had a significant dose-sparing effect when low doses were applied (12). Furthermore, mice vaccinated in all four limbs generated greater CD4+- and CD8+-specific T-cell responses compared to those of mice vaccinated only in the rear two limbs (12). In another phase I clinical study testing the safety and immunogenicity of plasmid DNA vaccines for Zika virus using I.M. injection delivered as a full dose at a single deltoid or as half doses injected in each deltoid showed antibodies delivered by split dose in different locations were two to three times greater than delivery in one location, and use of a needle-free system further improved the response (13). Our results are consistent with these preclinical and clinical studies on DNA vaccination delivery, showing multiple vaccination sites lead to better antigen-specific T-cell responses and anti-tumor immunity compared to single vaccination sites with the same total dosage.

We also observed an earlier and greater expression of encoded gene by DNA construct in I.D. delivery via customized needle-free biojector compared to I.M. delivery via customized needle-free biojector, showing a difference in kinetics between the different route of administration (see Fig. 2). This difference in the kinetics of expression may be attributed in part to the rapid turnover rate of epidermis, where the epidermis undergoes constant turnover as new cells are produced and old cells are replaced on the surface, as compared to the slow attrition and replacement of muscle cells (26). Because the DNA transfected basal skin cells rapidly rise through the epithelium, differentiate and then are sloughed off, the transduced cells are replaced by non-DNA transfected cells within 2 weeks (27). Therefore, the kinetics of the expression of marker DNA appear to peak earlier in skin (24 hours) and are gradually lost over time. We speculate that this may coincide with division and escape from the basement membrane of transduced basal keratinocytes, followed by a reduction in expression as these keratinocytes differentiate and die in the upper epithelial layers. In contrast, because the muscle does not have the same turnover rate and differentiation pattern as the skin, the expression of the DNA in transfected muscle occurs around 1 week after transfection and is sustained for a longer time (28). Therefore, the temporal pattern of expression of DNA delivered intradermally by the customized needle-free biojector is different from that delivered intramuscularly.

We further observed that I.M. delivery via the customized needle-free biojector appears to have enhanced expressions of encoded gene by DNA construct compared to I.M. delivery via conventional needle injection (see Fig. 2C). This observation could be due to the customized needle-free biojector having the ability to deliver the DNA construct into the cells along the path of the intradermal or intramuscular layer, as opposed to the targeted point of injection typical with conventional I.M. needle injection. This ability to deliver to cells along the layers of the path may occur from the greater hydrostatic pressure applied by the rapid force of the device’s injection process (as compared to relatively slow and gentle syringe injection), resulting in a wider distribution of the injected DNA construct (29). By distributing the DNA construct throughout the layers, a greater number of cells are more likely to undergo transfection, enhancing the overall gene expression. To gain further insight, it would be of interest in future investigations to employ histology examinations to compare the transfected cells along the delivery tract generated by the customized needle-free biojector or conventional needle devices.

We discovered that I.D. injection using the customized needle-free biojector leads to increased HPV-specific CD8+ T-cell responses and better survival of T-1-bearing mice as compared to I.M. injection using needle (see Fig. 3 and 4E). This may occur since the intradermal layer has a higher concentration of immature dendritic cells, known as Langerhans cells, compared to that of the intramuscular layer. Langerhans cells are a subtype of peripheral dendritic cells which play a crucial role in capturing, processing, and presenting antigenic material to naïve antigen-specific T cells (30). Langerhans cells exhibit migratory behavior as they transverse from the site of antigen uptake to present antigens to T cells (30). Studies have observed that Langerhans cells undergo a predetermined sequence of morphological changes in response to I.D. vaccine, resulting in a substantial migration of cells within and out of the epidermis (30). This highlights how intradermal immunization provides a unique advantage by specifically targeting follicular Langerhans cells, which have a preferential capacity to induce CD8+ T-cell responses that is not achievable through the intramuscular route (31). In addition, I.D. injection of vaccines has been shown to target dermal antigen-presenting cells, which mobilize both cellular and humoral arms of immunity (31). Collectively, the enrichment of Langerhans cells and dermal antigen-presenting cells in the intradermal layer offers a promising opportunity to introduce DNA directly into these specialized antigen-presenting cells, increasing the antigen-specific CD8+ T-cell responses. Nevertheless, the Stratis I.M. delivery device used clinically can deliver five times the volume (and thus dose) as compared to the Tropis I.D. delivery device. Therefore, Stratis delivery may induce a similar or even more potent response in patients, albeit using five times the amount of DNA vaccine. Furthermore, the Stratis device was safely used to deliver the VB10.16 DNA vaccine targeting HPV16 E6 and E7 in 34 subjects with HPV16+ CIN2/3 (32); mild to moderate injection site reactions were the most commonly reported adverse event (79%). HPV16 clearance was seen in 14 of 33 evaluable subjects. Reductions in lesion size were seen in 26 subjects, and 16 subjects had regression to CIN 0/1. Strong IFN-γ T-cell responses were correlated with lesion size reduction.

In this current study, we observed that I.D. delivery with the customized needle-free biojector showed an increased CD8+ T-cell immune responses compared to I.M. injection with a needle and syringe. However I.M. delivery with the customized needle-free biojector was similarly effective as I.M. injection with a needle and syringe in healthy mice. Previously, Alamri et al. has used the customized needle-free Tropis biojector to deliver SARS-CoV-2 DNA vaccines intradermally and intramuscularly (33, 34). Their studies have concluded that needle-free Tropis biojector delivery of the vaccine enhanced immunogenicity and reduced the doses needed to induce protective immunity in vaccinated mice (33). Furthermore, the DNA vaccine encoding SARS-CoV-2 spike subunit I protein was shown to elicit significant memory CD4+ and CD8+ T-cell responses when delivered by needle-free Tropis biojector (34). Taken together, these studies using a customized needle-free Tropis biojector from PharmaJet have shown promising results in mice and in humans, continuing to highlight its potential and significance in future clinical trials. Our preclinical study shows that the I.D. delivery of pBI-11 DNA vaccine through a customized needle-free Tropis biojector enhances gene expression, antigen-specific immune responses, and anti-tumor effects as compared to I.M. conventional needle injection. Importantly, this regimen was also well tolerated by mice, supporting the safety of delivery of pBI-11 DNA vaccine using this customized needle-free Tropis biojector in future clinical trials. Delivery I.M. via the Stratis device should also be considered because it can deliver five times higher doses than the Tropis device, and it was at least as effective as the conventional needle and syringe injections. Both the Tropis and Stratis devices have been used extensively in patients and have been well tolerated when delivering a diversity of vaccine types (13, 18).

## SUPPLEMENTAL MATERIAL

Supplemental File mbio.02121-23-s0001.pdf Figures S1 to S5 and Tables S1 to S6.

ASM does not own the copyrights to Supplemental Material that may be linked to, or accessed through, an article. The authors have granted ASM a non-exclusive, world-wide license to publish the Supplemental Material files. Please contact the corresponding author directly for reuse.
