# Supplementary material for: Immune responses, therapeutic anti-tumor effects, and tolerability upon therapeutic HPV16/18 E6/E7 DNA vaccination via needle-free biojector
Source: mBio. 2023 Oct 4;14(5):e02121-23. doi: 10.1128/mbio.02121-23 (PMC10653862; doi:10.1128/mbio.02121-23)
Supplement: Supplemental File — Figures S1 to S5 and Tables S1 to S6. [file mbio.02121-23-s0001.pdf]

Figure S1

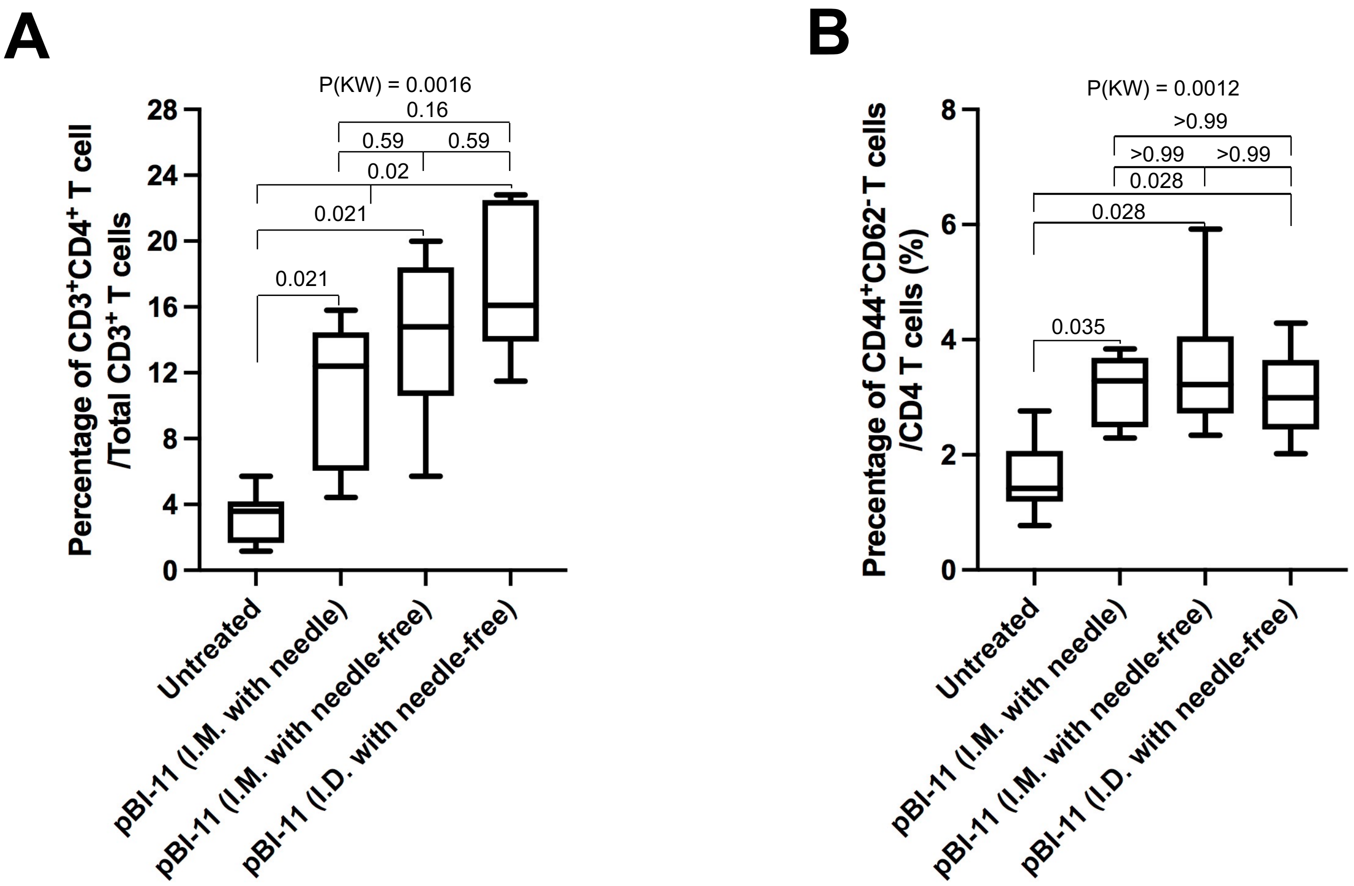
A
P(KW) = 0.0016
0.16
0.59
0.59
0.02
0.021
0.021
Percentage of CD3+CD4+ T cell /Total CD3+ T cells
28
24
20
16
12
8
4
0
Untreated
pBI-11 (I.M. with needle)
pBI-11 (I.M. with needle-free)
pBI-11 (I.D. with needle-free)
B
P(KW) = 0.0012
>0.99
>0.99
>0.99
0.028
0.028
0.035
Precentage of CD44+CD62- T cells /CD4 T cells (%)
8
6
4
2
0
Untreated
pBI-11 (I.M. with needle)
pBI-11 (I.M. with needle-free)
pBI-11 (I.D. with needle-free)

Figure S2

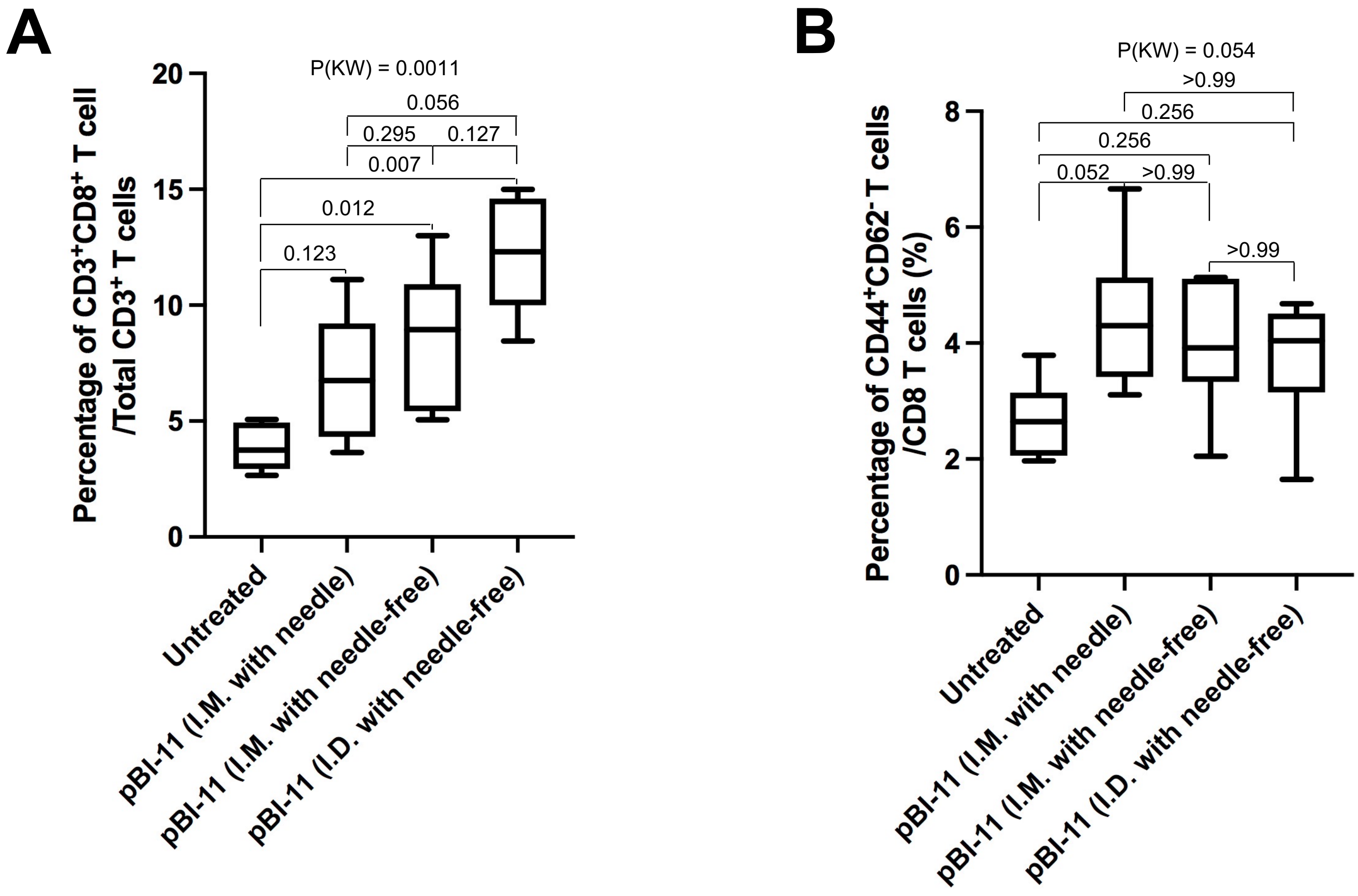
A
P(KW) = 0.0011
0.056
0.295
0.127
0.007
0.012
0.123
Percentage of CD3+CD8+ T cell /Total CD3+ T cells
20
15
10
5
0
Untreated
pBI-11 (I.M. with needle)
pBI-11 (I.M. with needle-free)
pBI-11 (I.D. with needle-free)
B
P(KW) = 0.054
>0.99
0.256
0.256
0.052
>0.99
>0.99
Percentage of CD44+CD62- T cells /CD8 T cells (%)
8
6
4
2
0
Untreated
pBI-11 (I.M. with needle)
pBI-11 (I.M. with needle-free)
pBI-11 (I.D. with needle-free)

Figure S3

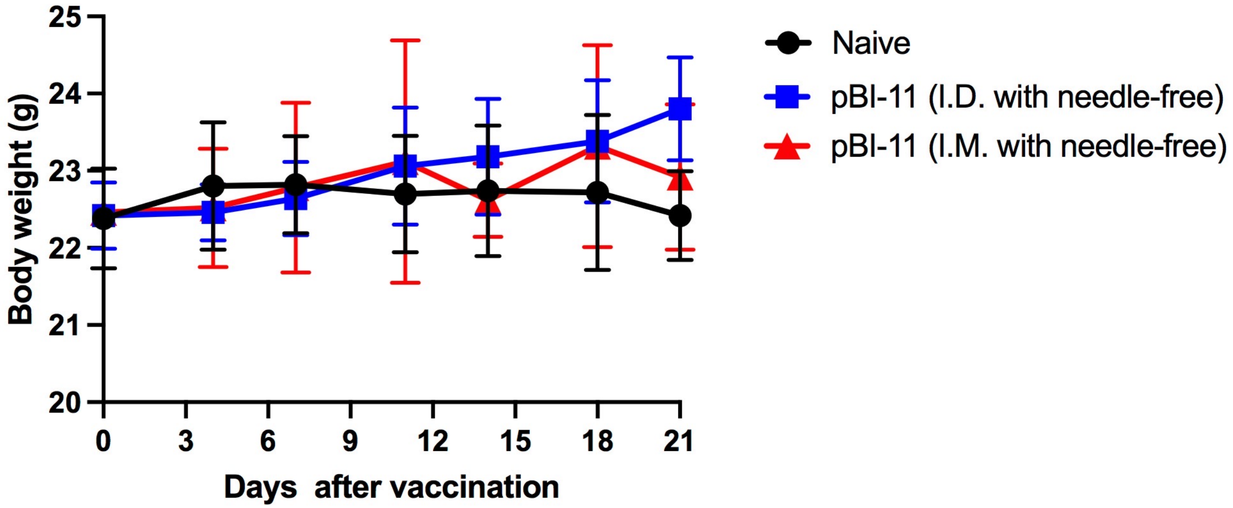

Body weight (g)
25
24
23
22
21
20
0
3
6
9
12
15
18
21
Days after vaccination
Naive
pBI-11 (I.D. with needle-free)
pBI-11 (I.M. with needle-free)

Figure S4

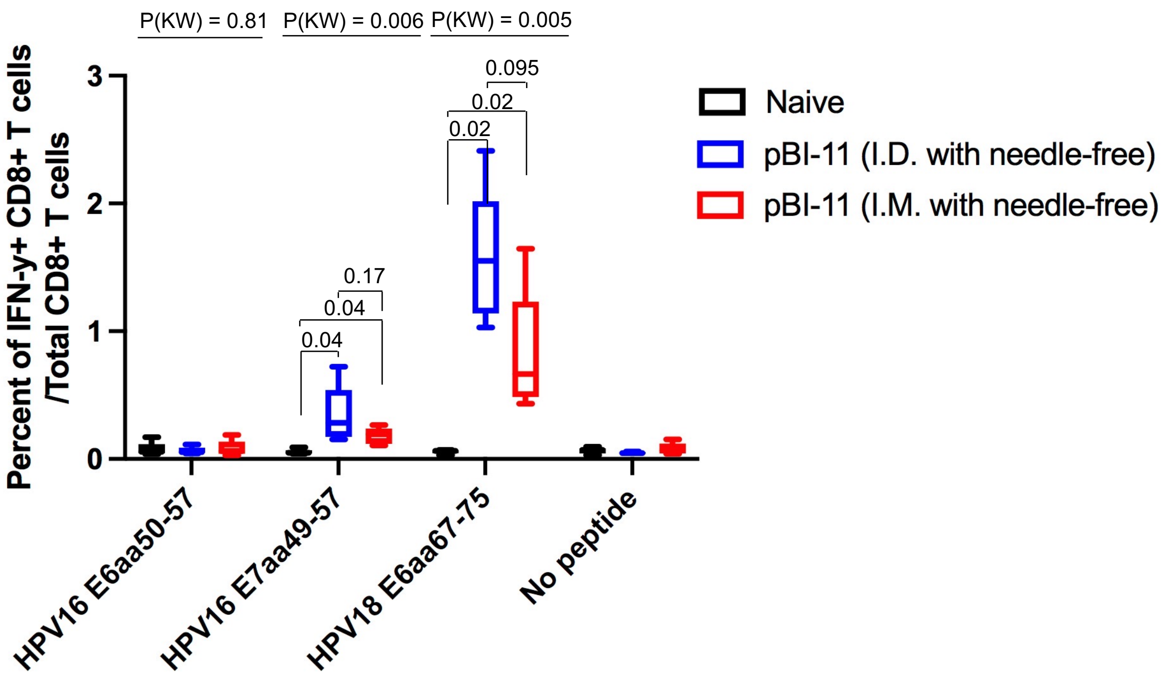
P(KW) = 0.81
P(KW) = 0.006
P(KW) = 0.005
0.095
0.02
0.02
0.17
0.04
0.04
Percent of IFN-γ+ CD8+ T cells /Total CD8+ T cells
3
2
1
0
HPV16 E6aa50-57
HPV16 E7aa49-57
HPV18 E6aa67-75
No peptide
Naive
pBI-11 (I.D. with needle-free)
pBI-11 (I.M. with needle-free)

Figure S5

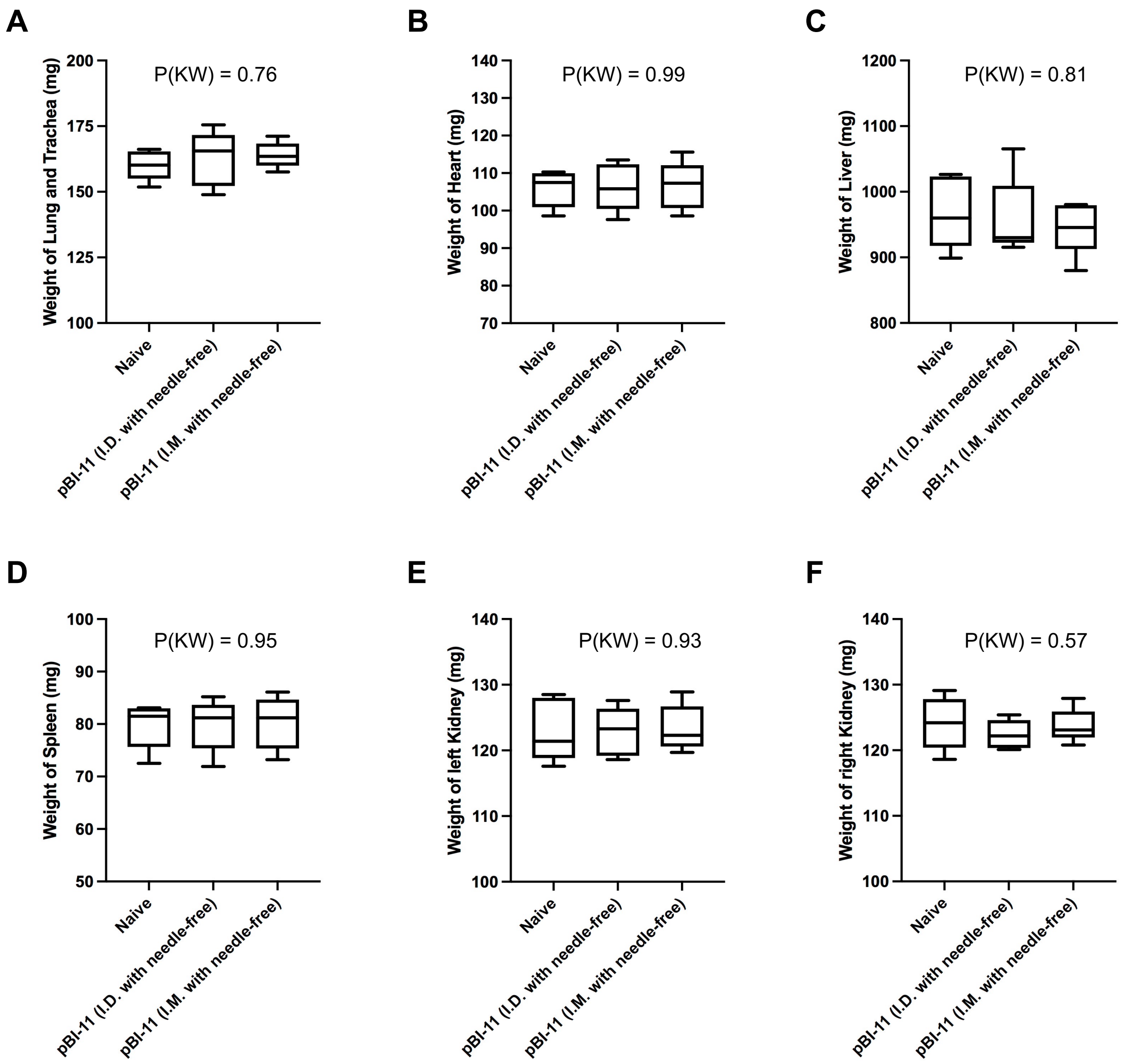
A
P(KW) = 0.76
Weight of Lung and Trachea (mg)
200
175
150
125
100
Naive
pBI-11 (I.D. with needle-free)
pBI-11 (I.M. with needle-free)
B
P(KW) = 0.99
Weight of Heart (mg)
140
130
120
110
100
90
80
70
Naive
pBI-11 (I.D. with needle-free)
pBI-11 (I.M. with needle-free)
C
P(KW) = 0.81
Weight of Liver (mg)
1200
1100
1000
900
800
Naive
pBI-11 (I.D. with needle-free)
pBI-11 (I.M. with needle-free)
D
P(KW) = 0.95
Weight of Spleen (mg)
100
90
80
70
60
50
Naive
pBI-11 (I.D. with needle-free)
pBI-11 (I.M. with needle-free)
E
P(KW) = 0.93
Weight of left Kidney (mg)
140
130
120
110
100
Naive
pBI-11 (I.D. with needle-free)
pBI-11 (I.M. with needle-free)
F
P(KW) = 0.57
Weight of right Kidney (mg)
140
130
120
110
100
Naive
pBI-11 (I.D. with needle-free)
pBI-11 (I.M. with needle-free)

Supplementary Figure Legends

Supplementary Figure 1. Distribution of CD4 T cells in TC-1 tumor-bearing B6 mice after pBI-11 DNA vaccination using needle or needle-free device. Female C57BL/6 mice (11- to 12-week-old, 6 – 8 mice/group) were injected with 2 $\times$ 10^5 of TC-1 cells subcutaneously on day 0. On day 3, the tumor-bearing mice were treated with pBI-11 DNA vaccination with a total of 50 μg of pBI-11 DNA (25 μg/50 μl/mouse per injection with two separate injections) through I.M. injection with needle, I.M. injection with needle-free device, or intradermal injection with needle-free device, under anesthesia with ketamine. The mice were boosted twice with the same dose and regimen at the indicated interval. One group of mice was left untreated as the control group. **A.** Percentages of $CD3^+$ $CD4^+$ T cells in periphery blood. **B.** Percentage of $CD44^+CD62L^-$ effector CD4 T cells in periphery blood.

Abbreviation: P(KW) - p-value based on Kruskal-Wallis test for global difference.

All other pair-wise p-values were from Wilcoxon test with multiplicity adjustment using Holm Method.

Supplementary Figure 2. Distribution of CD8 T cells in TC-1 tumor-bearing B6 mice after pBI-11 DNA vaccination using needle or needle-free device. Female C57BL/6 mice (11- to 12-week-old, 6 – 8 mice/group) were injected with 2 $\times$ 10^5 of TC-1 cells subcutaneously on day 0. On day 3, the tumor-bearing mice were treated with pBI-11 DNA vaccination with a total of 50 μg of pBI-11 DNA (25 μg/50 μl/mouse per injection with two separate injections) through I.M. injection with needle, I.M. injection with needle-free device, or intradermal injection using needle-free device, under anesthesia with ketamine.

The mice were boosted twice with the same dose and regimen at the indicated interval. One group of mice was left untreated as the control group. **A.** Percentages of $CD3^+$ $CD8^+$ T cells in periphery blood. **B**. Percentage of $CD44^+CD62L^-$ effector CD8 T cells in periphery blood.

Abbreviation: P(KW) - p-value based on Kruskal-Wallis test for global difference.

All other pair-wise p-values were from Wilcoxon test with multiplicity adjustment using Holm Method.

Supplementary Figure 3. Body weight of female C57BL/6 mice after vaccination. Female C57BL/6 mice (11- to 12-week-old, 5 mice/group) were divided into 3 groups. The mice of the first group (Naïve) were left unvaccinated. The mice of the second group were vaccinated with a total of 50 μg of pBI-11 DNA (25 μg/50 μl/mouse per injection with two separate injections) through I.D. injection on the back using a customized needle-free biojector under anesthesia with ketamine. The mice were boosted twice with the same regimen at a 1-week interval. The mice of the third group were vaccinated with a total of 50 μg of pBI-11 DNA (25 μg/50 μl/mouse per injection with two separate injections on each hind leg) through I.M. injection using a customized needle-free biojector under anesthesia with ketamine. The mice were boosted twice with the same regimen at a 1-week interval. The body weight of the mice was measured at an indicated time with a digital scale (from Ohaus Corporation, USA, model CS200).

Supplementary Figure 4. Analysis of HPV-specific CD8+ T cell responses of female C57BL/6 mice after vaccination. Female C57BL/6 mice (11- to 12-week-old, 5

mice/group) were divided into 3 groups. The mice of the first group (Naïve) were left unvaccinated. The mice of the second group were vaccinated with a total of 50 μg of pBI-11 DNA (25 μg/50 μl/mouse per injection with two separate injections) through I.D. injection on the back using a customized needle-free biojector under anesthesia with ketamine. The mice were boosted twice with the same regimen at a 1-week interval. The mice of the third group were vaccinated with a total of 50 μg of pBI-11 DNA (25 μg/50 μl/mouse per injection with two separate injections on each hind leg) through I.M. injection using a customized needle-free biojector under anesthesia with ketamine. The mice were boosted twice with the same regimen at a 1-week interval. 7 days after the last vaccination, splenocytes were prepared and stimulated with either HPV16 E6aa50-57 peptide (5 μg/ml), HPV16 E7aa49-57 peptide (1 μg/ml), or HPV18 E6aa67-75 peptide (1 μg/ml) in the presence of GolgiPlug (1 μl/ml) overnight. The cells were stained with PE-conjugated anti-mouse CD8a (clone 53.6.7) at 4˚C for 30 minutes. After washing, the cells were permeabilized and fixed with perm/fix buffer (from eBioscience) at 4˚C for 30 minutes. After washing, the cells were further stained with FITC-conjugated anti-mouse IFN-γ (clone XMG1.2) at 4˚C for 45 minutes. The cells were resuspended in PBS + 0.5% BSA after washing. The cells were acquired with FACSCalibur flow cytometer and data were analyzed with CellQuest Pro software.
Abbreviation: P(KW) - p-value based on Kruskal-Wallis test for global difference.
All other pair-wise p-values were from Wilcoxon test with multiplicity adjustment using Holm Method.

Supplementary Figure 5. Weight of organs of female C57BL/6 mice after vaccination. Female C57BL/6 mice (11- to 12-week-old, 5 mice/group) were divided into 3 groups. The mice of the first group (Naïve) were left unvaccinated. The mice of the second group were vaccinated with a total of 50 μg of pBI-11 DNA (25 μg/50 μl/mouse per injection with two separate injections) through I.D. injection on the back using a customized needle-free biojector anesthesia with ketamine. The mice were boosted twice with the same regimen at a 1-week interval. The mice of the third group were vaccinated with a total of 50 μg of pBI-11 DNA (25 μg/50 μl/mouse per injection with two separate injections on each hind leg) through I.M. injection using a customized needle-free biojector under anesthesia with ketamine. The mice were boosted twice with the same regimen at a 1-week interval. 7 days after the last vaccination, mice were sacrificed and lung with trachea (**A**), heart (**B**), liver (**C**), spleen (**D**), left kidney (**E**) and right kidney (**F**) were collected and weight was measured with a digital scale (from Mettler Toledo, Columbus, OH, USA, model AB104-S).

Abbreviation: P(KW) - p-value based on Kruskal-Wallis test for global difference.

Supplementary Tables

Table S1: Behavioral phenotype of mice receiving PBS, I.D., or I.M. vaccination treatment. The mice received vaccine injections on 03/30/2023, 04/6/2023 and 04/13/2023 with PBS, pBI-11 DNA delivered I.D. or I.M. using customized needle-free biojector. Mice were sacrificed 04/20/20/23. N (no) and 2 represent normal behaviors. Numerical rating scale 0-3.

Assessment of abnormal gait in vaccinated mice

Vaccination Group	Vaccination Mouse Number	Observation Date 3/29/23	Observation Date 4/1/23	Observation Date 4/5/23	Observation Date 4/8/23	Observation Date 4/12/23	Observation Date 4/15/23	Observation Date 4/18/23
PBS	PBS-1	N	N	N	N	N	N	N
	PBS-2	N	N	N	N	N	N	N
	PBS-3	N	N	N	N	N	N	N
	PBS-4	N	N	N	N	N	N	N
	PBS-5	N	N	N	N	N	N	N
ID	ID-1	N	N	N	N	N	N	N
	ID-2	N	N	N	N	N	N	N
	ID-3	N	N	N	N	N	N	N
	ID-4	N	N	N	N	N	N	N
	ID-5	N	N	N	N	N	N	N
IM	IM-1	N	N	N	N	N	N	N
	IM-2	N	N	N	N	N	N	N
	IM-3	N	N	N	N	N	N	N
	IM-4	N	N	N	N	N	N	N
	IM-5	N	N	N	N	N	N	N

N represents typical behavior.

Assessment of abnormal posture in vaccinated mice

Vaccination Group	Vaccination Mouse Number	Observation Date 3/29/23	Observation Date 4/1/23	Observation Date 4/5/23	Observation Date 4/8/23	Observation Date 4/12/23	Observation Date 4/15/23	Observation Date 4/18/23
PBS	PBS-1	N	N	N	N	N	N	N
	PBS-2	N	N	N	N	N	N	N
	PBS-3	N	N	N	N	N	N	N
	PBS-4	N	N	N	N	N	N	N
	PBS-5	N	N	N	N	N	N	N
ID	ID-1	N	N	N	N	N	N	N
	ID-2	N	N	N	N	N	N	N
	ID-3	N	N	N	N	N	N	N
	ID-4	N	N	N	N	N	N	N
	ID-5	N	N	N	N	N	N	N
IM	IM-1	N	N	N	N	N	N	N
	IM-2	N	N	N	N	N	N	N
	IM-3	N	N	N	N	N	N	N
	IM-4	N	N	N	N	N	N	N
	IM-5	N	N	N	N	N	N	N

N represents typical behavior.

Assessment of freezing behavior

Vaccination Group	Vaccination Mouse Number	Observation Date 3/29/23	Observation Date 4/1/23	Observation Date 4/5/23	Observation Date 4/8/23	Observation Date 4/12/23	Observation Date 4/15/23	Observation Date 4/18/23
PBS	PBS-1	N	N	N	N	N	N	N
	PBS-2	N	N	N	N	N	N	N
	PBS-3	N	N	N	N	N	N	N
	PBS-4	N	N	N	N	N	N	N
	PBS-5	N	N	N	N	N	N	N
ID	ID-1	N	N	N	N	N	N	N
	ID-2	N	N	N	N	N	N	N
	ID-3	N	N	N	N	N	N	N
	ID-4	N	N	N	N	N	N	N
	ID-5	N	N	N	N	N	N	N
IM	IM-1	N	N	N	N	N	N	N
	IM-2	N	N	N	N	N	N	N
	IM-3	N	N	N	N	N	N	N
	IM-4	N	N	N	N	N	N	N
	IM-5	N	N	N	N	N	N	N

N represents typical behavior.

Wild Running

Vaccination Group	Vaccination Mouse Number	Observation Date 3/29/23	Observation Date 4/1/23	Observation Date 4/5/23	Observation Date 4/8/23	Observation Date 4/12/23	Observation Date 4/15/23	Observation Date 4/18/23
PBS	PBS-1	N	N	N	N	N	N	N
	PBS-2	N	N	N	N	N	N	N
	PBS-3	N	N	N	N	N	N	N
	PBS-4	N	N	N	N	N	N	N
	PBS-5	N	N	N	N	N	N	N
ID	ID-1	N	N	N	N	N	N	N
	ID-2	N	N	N	N	N	N	N
	ID-3	N	N	N	N	N	N	N
	ID-4	N	N	N	N	N	N	N
	ID-5	N	N	N	N	N	N	N
IM	IM-1	N	N	N	N	N	N	N
	IM-2	N	N	N	N	N	N	N
	IM-3	N	N	N	N	N	N	N
	IM-4	N	N	N	N	N	N	N
	IM-5	N	N	N	N	N	N	N

N represents typical behavior.

Stereotypies

Vaccination Group	Vaccination Mouse Number	Observation Date 3/29/23	Observation Date 4/1/23	Observation Date 4/5/23	Observation Date 4/8/23	Observation Date 4/12/23	Observation Date 4/15/23	Observation Date 4/18/23
PBS	PBS-1	N	N	N	N	N	N	N
	PBS-2	N	N	N	N	N	N	N
	PBS-3	N	N	N	N	N	N	N
	PBS-4	N	N	N	N	N	N	N
	PBS-5	N	N	N	N	N	N	N
ID	ID-1	N	N	N	N	N	N	N
	ID-2	N	N	N	N	N	N	N
	ID-3	N	N	N	N	N	N	N
	ID-4	N	N	N	N	N	N	N
	ID-5	N	N	N	N	N	N	N
IM	IM-1	N	N	N	N	N	N	N
	IM-2	N	N	N	N	N	N	N
	IM-3	N	N	N	N	N	N	N
	IM-4	N	N	N	N	N	N	N
	IM-5	N	N	N	N	N	N	N

N represents typical behavior.

Escape

Vaccination Group	Vaccination Mouse Number	Observation Date 3/29/23	Observation Date 4/1/23	Observation Date 4/5/23	Observation Date 4/8/23	Observation Date 4/12/23	Observation Date 4/15/23	Observation Date 4/18/23
PBS	PBS-1	N	N	N	N	N	N	N
	PBS-2	N	N	N	N	N	N	N
	PBS-3	N	N	N	N	N	N	N
	PBS-4	N	N	N	N	N	N	N
	PBS-5	N	N	N	N	N	N	N
ID	ID-1	N	N	N	N	N	N	N
	ID-2	N	N	N	N	N	N	N
	ID-3	N	N	N	N	N	N	N
	ID-4	N	N	N	N	N	N	N
	ID-5	N	N	N	N	N	N	N
IM	IM-1	N	N	N	N	N	N	N
	IM-2	N	N	N	N	N	N	N
	IM-3	N	N	N	N	N	N	N
	IM-4	N	N	N	N	N	N	N
	IM-5	N	N	N	N	N	N	N

N represents typical behavior.

Exploring

Vaccination Group	Vaccination Mouse Number	Observation Date 3/29/23	Observation Date 4/1/23	Observation Date 4/5/23	Observation Date 4/8/23	Observation Date 4/12/23	Observation Date 4/15/23	Observation Date 4/18/23
PBS	PBS-1	2	2	2	2	2	2	2
	PBS-2	2	2	2	2	2	2	2
	PBS-3	2	2	2	2	2	2	2
	PBS-4	2	2	2	2	2	2	2
	PBS-5	2	2	2	2	2	2	2
ID	ID-1	2	2	2	2	2	2	2
	ID-2	2	2	2	2	2	2	2
	ID-3	2	2	2	2	2	2	2
	ID-4	2	2	2	2	2	2	2
	ID-5	2	2	2	2	2	2	2
IM	IM-1	2	2	2	2	2	2	2
	IM-2	2	2	2	2	2	2	2
	IM-3	2	2	2	2	2	2	2
	IM-4	2	2	2	2	2	2	2
	IM-5	2	2	2	2	2	2	2

2 represents normal behavior.

Digging

Vaccination Group	Vaccination Mouse Number	Observation Date 3/29/23	Observation Date 4/1/23	Observation Date 4/5/23	Observation Date 4/8/23	Observation Date 4/12/23	Observation Date 4/15/23	Observation Date 4/18/23
PBS	PBS-1	2	2	2	2	2	2	2
	PBS-2	2	2	2	2	2	2	2
	PBS-3	2	2	2	2	2	2	2
	PBS-4	2	2	2	2	2	2	2
	PBS-5	2	2	2	2	2	2	2
ID	ID-1	2	2	2	2	2	2	2
	ID-2	2	2	2	2	2	2	2
	ID-3	2	2	2	2	2	2	2
	ID-4	2	2	2	2	2	2	2
	ID-5	2	2	2	2	2	2	2
IM	IM-1	2	2	2	2	2	2	2
	IM-2	2	2	2	2	2	2	2
	IM-3	2	2	2	2	2	2	2
	IM-4	2	2	2	2	2	2	2
	IM-5	2	2	2	2	2	2	2

2 represents normal behavior.

Grooming

Vaccination Group	Vaccination Mouse Number	Observation Date 3/29/23	Observation Date 4/1/23	Observation Date 4/5/23	Observation Date 4/8/23	Observation Date 4/12/23	Observation Date 4/15/23	Observation Date 4/18/23
PBS	PBS-1	2	2	2	2	2	2	2
	PBS-2	2	2	2	2	2	2	2
	PBS-3	2	2	2	2	2	2	2
	PBS-4	2	2	2	2	2	2	2
	PBS-5	2	2	2	2	2	2	2
ID	ID-1	2	2	2	2	2	2	2
	ID-2	2	2	2	2	2	2	2
	ID-3	2	2	2	2	2	2	2
	ID-4	2	2	2	2	2	2	2
	ID-5	2	2	2	2	2	2	2
IM	IM-1	2	2	2	2	2	2	2
	IM-2	2	2	2	2	2	2	2
	IM-3	2	2	2	2	2	2	2
	IM-4	2	2	2	2	2	2	2
	IM-5	2	2	2	2	2	2	2

2 represents normal behavior.

Rearing

Vaccination Group	Vaccination Mouse Number	Observation Date 3/29/23	Observation Date 4/1/23	Observation Date 4/5/23	Observation Date 4/8/23	Observation Date 4/12/23	Observation Date 4/15/23	Observation Date 4/18/23
PBS	PBS-1	2	2	2	2	2	2	2
	PBS-2	2	2	2	2	2	2	2
	PBS-3	2	2	2	2	2	2	2
	PBS-4	2	2	2	2	2	2	2
	PBS-5	2	2	2	2	2	2	2
ID	ID-1	2	2	2	2	2	2	2
	ID-2	2	2	2	2	2	2	2
	ID-3	2	2	2	2	2	2	2
	ID-4	2	2	2	2	2	2	2
	ID-5	2	2	2	2	2	2	2
IM	IM-1	2	2	2	2	2	2	2
	IM-2	2	2	2	2	2	2	2
	IM-3	2	2	2	2	2	2	2
	IM-4	2	2	2	2	2	2	2
	IM-5	2	2	2	2	2	2	2

2 represents normal behavior.

Table S2: Assessment for eschar formation at the vaccinated site

		**Time after 1st Vaccination (03/30/2023)**		**Time after 2nd Vaccination (04/06/2023)**		**Time after 3rd Vaccination (04/13/2023)**	
Vaccination Group	Vaccination Mouse Number	2 hours	24 hours	2 hours	24 hours	2 hours	24 hours
ID	ID-1	0	0	0	0	0	0
	ID-2	0	0	0	0	0	0
	ID-3	0	0	0	0	0	0
	ID-4	0	0	0	0	0	0
	ID-5	0	0	0	0	0	0
IM	IM-1	0	0	0	0	0	0
	IM-2	0	0	0	0	0	0
	IM-3	0	0	0	0	0	0
	IM-4	0	0	0	0	0	0
	IM-5	0	0	0	0	0	0

0- Within normal limits / No significant findings (unremarkable)

Table S3: Assessment of edema formation at vaccinated site

		**Time after 1st Vaccination (03/30/2023)**		**Time after 2nd Vaccination (04/06/2023)**		**Time after 3rd Vaccination (04/13/2023)**	
Vaccination Group	Vaccination Mouse Number	2 hours	24 hours	2 hours	24 hours	2 hours	24 hours
ID	ID-1	0	0	0	0	0	0
	ID-2	0	0	0	0	0	0
	ID-3	0	0	0	0	0	0
	ID-4	0	0	0	0	0	0
	ID-5	0	0	0	0	0	0
IM	IM-1	0	0	0	0	0	0
	IM-2	0	0	0	0	0	0
	IM-3	0	0	0	0	0	0
	IM-4	0	0	0	0	0	0
	IM-5	0	0	0	0	0	0

0 – Within normal limits / No significant findings (unremarkable)

Table S4: Complete blood count of vaccinated mice.

Mouse Number	1	2	3	4	5	6	7	8	9	10	11	12	13	14	15
Test Day	04/19/2023	04/19/2023	04/19/2023	04/19/2023	04/19/2023	04/19/2023	04/19/2023	04/19/2023	04/19/2023	04/19/2023	04/19/2023	04/19/2023	04/19/2023	04/19/2023	04/19/2023
Species	Mouse	Mouse	Mouse	Mouse	Mouse	Mouse	Mouse	Mouse	Mouse	Mouse	Mouse	Mouse	Mouse	Mouse	Mouse
Strain	C57BL/6	C57BL/6	C57BL/6	C57BL/6	C57BL/6	C57BL/6	C57BL/6	C57BL/6	C57BL/6	C57BL/6	C57BL/6	C57BL/6	C57BL/6	C57BL/6	C57BL/6
Age (week)	20	20	20	20	20	20	20	20	20	20	20	20	20	20	20
Sex	Female	Female	Female	Female	Female	Female	Female	Female	Female	Female	Female	Female	Female	Female	Female
Vaccination Group Number	PBS-1	PBS-2	PBS-3	PBS-4	PBS-5	ID-1	ID-2	ID-3	ID-4	ID-5	IM-1	IM-2	IM-3	IM-4	IM-5
RBC (M/uL)	11.53	10.32	10.22	10.39	10.11	10.08	9.97	11.02	10.62	10.53	10.53	10.59	10.36	9.88	10.79
HGB (g/dL)	17.5	15.9	15.8	15.5	15.2	14.9	15.0	16.5	16.0	15.8	15.9	16.1	15.6	15.0	16.3
HCT (%)	55.8	50.2	49.5	49.9	48.5	47.5	46.8	52.2	50.2	49.8	49.9	51.0	49.6	47.3	51.1
MCV (fL)	48.4	48.6	48.4	48.0	48.0	47.1	46.9	47.4	47.3	47.3	47.4	48.2	47.9	47.9	47.4
MCH (pg)	15.2	15.4	15.5	14.9	15.0	14.8	15.0	15.0	15.1	15.0	15.1	15.2	15.1	15.2	15.1
MCHC (g/dL)	31.4	31.7	31.9	31.1	31.3	31.4	32.1	31.6	31.9	31.7	31.9	31.6	31.5	31.7	31.9
RDW-SD (fL)	28.7	28.2	28.3	28.9	28.2	27.1	28.1	27.2	28.4	26.9	28.9	28.3	28.2	27.4	26.9
RDW-CV (%)	25.1	23.5	23.3	24.0	23.5	23.3	24.0	24.3	24.4	23.8	24.7	24.1	23.8	23.3	23.9
RET (K/ul)	475.0	370.5	365.9	354.3	349.8	294.3	349.9	412.1	431.2	391.7	360.1	517.9	500.4	461.4	428.4
IRF (%)	56.2	56.4	58.5	59.4	55.6	52.3	61.1	55.2	60.5	51.8	54.6	61.5	59.6	59.8	52.7
LFR (%)	43.8	43.6	41.5	40.6	44.4	47.7	38.9	44.8	39.5	48.2	45.4	38.5	40.4	40.2	47.3
MFR (%)	17.8	18.7	19.5	19.7	21.0	19.7	20.0	21.8	21.1	22.5	17.3	18.6	18.9	17.3	21.2
HFR (%)	38.4	37.7	39.0	39.7	34.6	32.6	41.1	33.4	39.4	29.3	37.3	42.9	40.7	42.5	31.8
RET-He (pg)	17.5	17.4	17.2	17.1	17.1	16.8	17.4	16.7	17.1	16.6	17.4	17.7	17.7	17.7	16.7
PLT (K/uL)	1159	885	707	953	900	914	834	1150	981	1032	860	1165	1095	1119	1064
PDW (fL)	7.3	6.6	6.8	6.7	6.5	6.6	6.6	6.8	6.7	6.5	6.8	6.7	6.7	6.8	6.6
MPV (fL)	8.2	8.0	8.2	8.1	8.1	8.0	8.2	8.0	7.8	7.9	8.2	8.0	7.9	8.0	8.0
P-LCR (%)	2.4	1.6	3.6	1.6	2.0	3.1	2.6	1.9	1.6	2.7	3.0	1.7	2.5	2.6	2.0
PCT (%)	0.95	0.71	0.58	0.77	0.73	0.73	0.68	0.92	0.77	0.82	0.71	0.93	0.87	0.90	0.85
WBC (K/uL)	12.23	9.45	10.16	10.70	7.30	3.01	3.77	11.55	14.36	5.03	6.13	8.85	9.65	7.94	6.75
NEUT (K/uL)	1.07	0.84	1.49	1.39	0.57	0.31	0.26	0.91	0.68	0.40	0.52	0.67	0.65	0.23	0.72
NEUT (%)	8.7	8.9	14.6	13.0	7.8	10.3	6.9	7.8	4.7	7.9	8.7	7.6	6.7	2.9	10.7
LYMPH (K/uL)	10.83	8.3	8.37	8.99	6.52	2.59	3.37	10.31	13.38	4.47	5.38	7.94	8.77	7.44	5.86
LYMPH (%)	88.6	87.8	82.4	84.0	89.3	86.0	89.4	89.3	93.2	88.9	87.8	89.7	90.9	93.7	86.8

MONO (K/uL)	0.11	0.13	0.06	0.06	0.07	0.03	0.06	0.07	0.06	0.02	0.07	0.09	0.08	0.11	0.03
MONO (%)	0.9	1.4	0.6	0.6	1.0	1.0	1.6	0.6	0.4	0.4	1.1	1.0	0.8	1.4	0.4
EO (K/uL)	0.22	0.18	0.24	0.26	0.14	0.08	0.08	0.26	0.23	0.14	0.15	0.15	0.15	0.16	0.14
EO (%)	1.8	1.9	2.4	2.4	1.9	2.7	2.1	2.3	1.6	2.8	2.4	1.7	1.6	2.0	2.1
BASO (K/uL)	0.00	0.00	0.00	0.00	0.00	0.00	0.00	0.00	0.01	0.00	0.00	0.00	0.00	0.00	0.00
BASO (%)	0.0	0.0	0.0	0.0	0.0	0.0	0.0	0.0	0.1	0.0	0.0	0.0	0.0	0.0	0.0

Summary of complete blood count studies in vaccinated mice using serum one week after final vaccination
Abbreviations: RBC = Red Blood Cell Count; HGB = Hemoglobin value; HCT = Hematocrit value; MCV = Mean Corpuscular Volume; MHC = Mean Corpuscular Hemoglobin; MCHC = Mean Corpuscular Hemoglobin Concentration; RDW-SD = Red Cell Distribution Width Standard Deviation; RDW-CV = Red Cell Distribution Width Coefficient of Variation; RET = Reticulocytes; IRF =Immature Reticulocyte Fraction; LFR = Low Fluorescence Ratio; MFR = Medium Fluorescence Ratio; HFR = High Fluorescence Ratio; RET-He = Retic Hemoglobin; PLT = Platelet count; PDW = Platelet Distribution Width; MPV = Mean Platelet Volume ; P-LCR = Platelet Large Cell Ratio; PCT = Plateletcrit value; WBC = White Blood Cell Count; NEUT = Neutrophil percent; LYMPH = Lymphocyte percent; MONO = Monocyte; EO = Eosinophil; BASO = Basophil

Table S5: Summary of the biochemistry study in vaccinated mice

Mouse Number	1	2	3	4	5	6	7	8	9	10	11	12	13	14	15
Test Day	04/19/2023	04/19/2023	04/19/2023	04/19/2023	04/19/2023	04/19/2023	04/19/2023	04/19/2023	04/19/2023	04/19/2023	04/19/2023	04/19/2023	04/19/2023	04/19/2023	04/19/2023
Species	Mouse	Mouse	Mouse	Mouse	Mouse	Mouse	Mouse	Mouse	Mouse	Mouse	Mouse	Mouse	Mouse	Mouse	Mouse
Strain	C57BL/6	C57BL/6	C57BL/6	C57BL/6	C57BL/6	C57BL/6	C57BL/6	C57BL/6	C57BL/6	C57BL/6	C57BL/6	C57BL/6	C57BL/6	C57BL/6	C57BL/6
Age (week)	20	20	20	20	20	20	20	20	20	20	20	20	20	20	20
Sex	Female	Female	Female	Female	Female	Female	Female	Female	Female	Female	Female	Female	Female	Female	Female
Vaccination Group Number	ID-1	ID-2	ID-3	ID-4	ID-5	IM-1	IM-2	IM-3	IM-4	IM-5	PBS-1	PBS-2	PBS-3	PBS-4	PBS-5
ALB (g/dL)	3.5	3.3	3.3	3.3	3.3	3.4	3.3	3.4	3.3	3.4	3.3	3.3	3.2	3.2	3.4
ALB/GLOB ratio	1.8	1.7	1.8	1.8	1.8	1.7	1.8	1.7	1.7	1.8	1.8	1.7	1.9	1.8	1.9
ALP (U/L)	113	116	108	100	90	103	85	109	112	101	135	112	111	116	100
ALT (U/L)	27	25	40	26	25	28	20	23	23	24	24	25	27	26	27
AST (U/L)	58	45	49	45	43	53	41	46	47	49	45	49	62	55	53
BUN (mg/dL)	23	21	22	21	19	23	18	17	24	22	24	22	24	23	22
BUN/CREA ratio	230.0	70.00	110.0	105.0	95.0	115.0	180.0	85.0	120.0	110.0	120.0	110.0	120.0	115.0	110.0
CA (mg/dL)	9.0	9.1	8.9	9.0	9.2	8.8	8.9	8.8	9.0	9.3	9.1	8.8	9.3	9.1	8.9
CHOL (mg/dL)	114	116	112	108	106	108	110	126	102	115	102	105	103	103	107
CK (U/L)	89	124	149	195	96	120	50	349	293	246	91	145	660	334	95
CL (mmol/L)	110	111	110	111	109	112	111	112	113	112	113	111	113	112	110
CREA (mg/dL)	0.1	0.3	0.2	0.2	0.2	0.2	0.1	0.2	0.2	0.2	0.2	0.2	0.2	0.2	0.2
GLOBULIN (g/dL)	2.0	2.0	1.8	1.8	1.8	2.0	1.8	2.0	1.9	1.9	1.8	1.9	1.7	1.8	1.8
GLUC (mg/dL)	165	174	165	174	184	155	183	212	176	177	177	161	222	221	162
K (mmol/L)	5.6	4.7	5.0	4.7	4.7	5.1	5.1	5.0	4.9	5.0	5.3	5.0	6.1	5.5	5.1
NA (mmol/L)	150	153	150	149	150	153	150	151	151	152	155	153	152	152	152
NA/K ratio	27	33	30	32	32	30	29	30	31	30	29	31	25	28	30
PHOS (mg/dL)	5.5	5.1	5.7	4.9	4.7	5.1	5.9	6.0	5.4	5.6	5.4	5.4	5.5	5.3	5.3
TBIL (mg/dL)	0.3	0.2	0.2	0.2	0.2	0.3	0.2	0.2	0.2	0.2	0.2	0.2	0.2	0.2	0.2
TP (g/dL)	5.5	5.3	5.1	5.1	5.1	5.4	5.1	5.4	5.2	5.3	5.1	5.2	4.9	5.0	5.2

Summary of biochemistry study in vaccinated mice using serum one week after final vaccination

Abbreviations: ALB = Albumin; ALB/GLOB = Albumin to Globulin; ALP = Alkaline phosphatase; ALT = Alanine Aminotransferase; AST = Aspartate Aminotransferase; BUN = Blood Urea Nitrogen; BUN/CREA = Blood Urea Nitrogen to Creatinine; CA = Calcium; CHOL = Cholesterol; CK = Creatine Kinase; CL = Chloride; CREA = Creatinine; GLUC = Glucose; K = Potassium; NA = Sodium; TBIL = Total Bilrubin; TP = Total Protein;

Table S6 Histological examination of key organs in mice receiving vaccination. (Mice were sacrificed on 04/20/2023).

Vaccination Group	PBS				
Mouse Vaccination Number:	PBS-1	PBS-2	PBS-3	PBS-4	PBS-5
Date	04/20/2023	04/20/2023	04/20/2023	04/20/2023	04/20/2023
Brain	WNL/NSF	WNL/NSF	WNL/NSF	WNL/NSF	WNL/NSF
Thymus	WNL/NSF	WNL/NSF	WNL/NSF	WNL/NSF	WNL/NSF
Lung	WNL/NSF	WNL/NSF	WNL/NSF	WNL/NSF	WNL/NSF
Trachea	WNL/NSF	WNL/NSF	WNL/NSF	WNL/NSF	WNL/NSF
Heart	WNL/NSF	WNL/NSF	WNL/NSF	WNL/NSF	WNL/NSF
Stomach	WNL/NSF	WNL/NSF	WNL/NSF	WNL/NSF	WNL/NSF
Small intestine	WNL/NSF	WNL/NSF	WNL/NSF	WNL/NSF	WNL/NSF
Large intestine	WNL/NSF	WNL/NSF	WNL/NSF	WNL/NSF	WNL/NSF
Pancreas	WNL/NSF	WNL/NSF	WNL/NSF	WNL/NSF	WNL/NSF
Liver	WNL/NSF	WNL/NSF	WNL/NSF	WNL/NSF	WNL/NSF
Right kidney	WNL/NSF	WNL/NSF	WNL/NSF	WNL/NSF	WNL/NSF
Left kidney	WNL/NSF	WNL/NSF	WNL/NSF	WNL/NSF	WNL/NSF
Ovaries	WNL/NSF	WNL/NSF	WNL/NSF	WNL/NSF	WNL/NSF
Fallopian Tubes	WNL/NSF	WNL/NSF	WNL/NSF	WNL/NSF	WNL/NSF
Uterus	WNL/NSF	WNL/NSF	WNL/NSF	WNL/NSF	WNL/NSF
Adrenal glands	WNL/NSF	WNL/NSF	WNL/NSF	WNL/NSF	WNL/NSF
Spleen	NT	WNL/NSF	WNL/NSF	WNL/NSF	WNL/NSF

WNL/NSF = Within normal limits / No significant findings (unremarkable)
NT = No Tissue

Vaccination Group	pBI-11 DNA delivered ID by Tropis				
Mouse Vaccination Number:	ID-1	ID-2	ID- 3	ID- 4	ID- 5
Date	04/20/2023	04/20/20223	04/20/2023	04/20/2023	04/20/2023
Brain	WNL/NSF	WNL/NSF	WNL/NSF	WNL/NSF	WNL/NSF
Thymus	WNL/NSF	WNL/NSF	WNL/NSF	WNL/NSF	WNL/NSF
Lung	WNL/NSF	WNL/NSF	WNL/NSF	WNL/NSF	WNL/NSF
Trachea	WNL/NSF	WNL/NSF	WNL/NSF	WNL/NSF	WNL/NSF
Heart	WNL/NSF	WNL/NSF	WNL/NSF	WNL/NSF	WNL/NSF
Stomach	WNL/NSF	WNL/NSF	WNL/NSF	WNL/NSF	WNL/NSF
Small intestine	WNL/NSF	WNL/NSF	WNL/NSF	WNL/NSF	WNL/NSF
Large intestine	WNL/NSF	WNL/NSF	WNL/NSF	WNL/NSF	WNL/NSF
Pancreas	WNL/NSF	WNL/NSF	WNL/NSF	WNL/NSF	WNL/NSF
Liver	WNL/NSF	WNL/NSF	WNL/NSF	WNL/NSF	WNL/NSF

Right kidney	WNL/NSF	WNL/NSF	WNL/NSF	WNL/NSF	WNL/NSF
Left kidney	WNL/NSF	WNL/NSF	WNL/NSF	WNL/NSF	WNL/NSF
Ovaries	WNL/NSF	WNL/NSF	WNL/NSF	WNL/NSF	WNL/NSF
Fallopian Tubes	WNL/NSF	WNL/NSF	WNL/NSF	WNL/NSF	WNL/NSF
Uterus	NT	WNL/NSF	WNL/NSF	WNL/NSF	WNL/NSF
Adrenal glands	WNL/NSF	WNL/NSF	NT	WNL/NSF	WNL/NSF
Spleen	WNL/NSF	WNL/NSF	WNL/NSF	NT	WNL/NSF

WNL/NSF = Within normal limits / No significant findings (unremarkable)
NT = No Tissue

Vaccination Group	pBI-11 DNA delivered IM by Tropis				
Mouse Vaccination Number:	IM-1	IM- 2	IM- 3	IM-4	IM-5
Date	4/20/2023	4/20/2023	4/20/2023	4/20/2023	4/20/2023
Brain	WNL/NSF	WNL/NSF	WNL/NSF	WNL/NSF	WNL/NSF
Thymus	WNL/NSF	WNL/NSF	WNL/NSF	WNL/NSF	WNL/NSF
Lung	WNL/NSF	WNL/NSF	WNL/NSF	WNL/NSF	WNL/NSF
Trachea	WNL/NSF	WNL/NSF	WNL/NSF	WNL/NSF	WNL/NSF
Heart	WNL/NSF	WNL/NSF	WNL/NSF	WNL/NSF	WNL/NSF
Stomach	WNL/NSF	WNL/NSF	WNL/NSF	WNL/NSF	WNL/NSF
Small intestine	WNL/NSF	WNL/NSF	WNL/NSF	WNL/NSF	WNL/NSF
Large intestine	WNL/NSF	WNL/NSF	WNL/NSF	WNL/NSF	WNL/NSF
Pancreas	WNL/NSF	WNL/NSF	WNL/NSF	WNL/NSF	WNL/NSF
Liver	WNL/NSF	WNL/NSF	WNL/NSF	WNL/NSF	WNL/NSF
Right kidney	WNL/NSF	WNL/NSF	WNL/NSF	WNL/NSF	WNL/NSF
Left kidney	WNL/NSF	WNL/NSF	WNL/NSF	WNL/NSF	WNL/NSF
Ovaries	WNL/NSF	WNL/NSF	WNL/NSF	WNL/NSF	WNL/NSF
Fallopian Tubes	WNL/NSF	WNL/NSF	WNL/NSF	WNL/NSF	WNL/NSF
Uterus	WNL/NSF	WNL/NSF	WNL/NSF	WNL/NSF	WNL/NSF
Adrenal glands	WNL/NSF	WNL/NSF	WNL/NSF	WNL/NSF	WNL/NSF
Spleen	WNL/NSF	WNL/NSF	WNL/NSF	WNL/NSF	WNL/NSF

WNL/NSF = Within normal limits / No significant findings (unremarkable)
NT = No Tissue
